# Maternal vitamin D during pregnancy and offspring autism and autism-associated traits: a prospective cohort study

**DOI:** 10.1186/s13229-022-00523-4

**Published:** 2022-11-12

**Authors:** Paul Madley-Dowd, Christina Dardani, Robyn E. Wootton, Kyle Dack, Tom Palmer, Rupert Thurston, Alexandra Havdahl, Jean Golding, Deborah Lawlor, Dheeraj Rai

**Affiliations:** 1 Centre for Academic Mental Health, Population Health Sciences, Bristol Medical School, University of Bristol, Oakfield House, Oakfield Grove, Bristol, BS8 2BN UK; 2 MRC Integrative Epidemiology Unit, University of Bristol, Bristol, UK; 3 Population Health Sciences, Bristol Medical School, University of Bristol, Bristol, UK; 4 Nic Waals Institute, Lovisenberg Diaconal Hospital, Oslo, Norway; 5 Swansea Bay University Health Board, Port Talbot, UK; 6 Department of Psychology, PROMENTA Research Center, University of Oslo, Oslo, Norway; 7 Department of Mental Disorders, Norwegian Institute of Public Health, Oslo, Norway; 8 Centre for Academic Child Health, Population Health Sciences, Bristol Medical School, University of Bristol, Bristol, UK; 9 Avon and Wiltshire Partnership, NHS Mental Health Trust, Bristol, UK

**Keywords:** ALSPAC, Autism, Mendelian randomization, Pregnancy, Vitamin D

## Abstract

**Background:**

There has been a growing interest in the association between maternal levels of vitamin D during pregnancy and offspring autism. However, whether any associations reflect causal effects is still inconclusive.

**Methods:**

We used data from a UK-based pregnancy cohort study (Avon Longitudinal Study of Parents and Children) comprising 7689 births between 1991 and 1992 with maternal blood vitamin D levels recorded during pregnancy and at least one recorded outcome measure, including autism diagnosis and autism-associated traits. The association between each outcome with seasonal and gestational age-adjusted maternal serum 25-hydroxyvitamin D during pregnancy was estimated using confounder-adjusted regression models. Multiple imputation was used to account for missing data, and restricted cubic splines were used to investigate nonlinear associations. Mendelian randomization was used to strengthen causal inference.

**Results:**

No strong evidence of an association between maternal serum 25-hydroxyvitamin D during pregnancy and any offspring autism-associated outcome was found using multivariable regression analysis (autism diagnosis: adjusted OR = 0.98, 95% CI = 0.90–1.06), including with multiple imputation (autism diagnosis: adjusted OR = 0.99, 95% CI = 0.93–1.06), and no evidence of a causal effect was suggested by Mendelian randomization (autism diagnosis: causal OR = 1.08, 95% CI = 0.46–2.55). Some evidence of increased odds of autism-associated traits at lower levels of maternal serum 25-hydroxyvitamin D was found using spline analysis.

**Limitations:**

Our study was potentially limited by low power, particularly for diagnosed autism cases as an outcome. The cohort may not have captured the extreme lows of the distribution of serum 25-hydroxyvitamin D, and our analyses may have been biased by residual confounding and missing data.

**Conclusions:**

The present study found no strong evidence of a causal link between maternal vitamin D levels in pregnancy and offspring diagnosis or traits of autism.

**Supplementary Information:**

The online version contains supplementary material available at 10.1186/s13229-022-00523-4.

## Background

The association between maternal vitamin D levels during pregnancy and offspring risk of developing autism has garnered increasing attention in the past few years [1–6]. This association has been of particular interest due to the plausibility of several potential modifiable mechanisms of action. These include the involvement of increased vitamin D levels in the reduction in neuro-inflammation, upregulation of antioxidants and specific regulation of serotonin in different regions of the brain. All of the above have been suggested to be different between individuals with autism and the neurotypical population [1, 2]. Vitamin D is a neuroactive hormone, with evidence accumulating to suggest it is involved in neurodevelopment more generally via pathways that include the expression of genes influencing neuronal differentiation, structure and metabolism, as well as the expression of neurotrophic factor, cytokine regulation, neurotransmitter synthesis and intracellular calcium signalling [7].

Systematic reviews of the available epidemiological evidence [3–5] have collectively assessed 11 studies investigating an association between vitamin D in pregnancy and offspring autism, seven of which measured vitamin D using maternal serum 25-hydroxyvitamin D (25(OH)D) [8–14], two using neonatal blood spots [15, 16], and the remaining two using maternal lifetime diagnosis of vitamin D deficiency [17] and maternal supplementation [18], respectively. These studies were most often cohort or case–control studies, frequently with low sample sizes.

Risk of bias was assessed in each of the reviews using the Newcastle Ottawa Scale [19] which suggested that most studies were of high quality, though there was disagreement between the reviews. It has been argued that suitable control for confounding is not well defined in the Newcastle Ottawa Scale [20]. Nine of the studies controlled for all or most of the key confounders including maternal socio-economic position, age, ethnicity, parity, body mass index (BMI) and smoking in pregnancy [8–14, 16, 17]; two studies made no adjustment [15, 18]. As common with observational studies, there was the potential for residual or unmeasured confounding. To account for this, two studies made use of exposure-discordant sibling comparisons, concluding that associations could not entirely be accounted for by confounding shared between siblings [13, 15]. Several studies adjusted for post-exposure variables, notably gestational age at birth and birthweight, which may lie on the causal pathway. Increased odds of autism among those with lower levels of maternal vitamin D were suggested by the two meta-analyses conducted [3, 4]. Publication bias, whereby studies finding null associations are less likely to be published [21, 22], was not suggested to be driving the association.

Several other recent studies not included in these reviews have been published. Sourander et al. used a nested case–control study of singleton live births in Finland from 1987 to 2004 to show that there were increased adjusted odds of autism in lower quintiles of maternal vitamin D levels as compared to the highest quintile [23]. Windham et al.’s case–control study explored nonlinear patterns and further investigated potentially vulnerable subgroups [24]. No strong evidence for an association between vitamin D in pregnancy and offspring risk of autism was found; however, this study did provide evidence for interaction effects that suggested protective effects of higher vitamin D levels in some subgroups (non-Hispanic Whites, males). They further provided evidence of a j-shape curve using restricted cubic splines such that both low and high levels of vitamin D may reduce the odds for autism, though the authors stated the need for more evidence regarding nonlinear associations. Finally, Guerini et al. compared the distribution of single nucleotide polymorphisms (SNPs) for vitamin D receptors among 100 Italian children with autism and their siblings without autism [25]. They found a skew in the distribution of the FokI genotype among children with autism as compared to their siblings without autism suggesting a potential mechanism of action.

Triangulation of evidence across studies with different key sources of bias can help improve causal understanding [26, 27]. The current evidence base consists largely of observational analyses and a small number of exposure-discordant sibling studies. Other methods such as Mendelian randomisation (MR), an instrumental variable technique employing genetic variants as a proxy for the exposure, will provide further evidence as to whether the association reflects a causal effect. Further exploration of nonlinear associations is also needed to explore whether both extreme low and high levels of vitamin D during pregnancy are associated with changes in the risk of offspring autism. The current study aimed to i) perform observational analyses of prospectively collected maternal blood vitamin D during pregnancy and offspring risk of autism diagnosis and autism-associated traits in the Avon Longitudinal Study of Parents and Children (ALSPAC) in order to replicate the findings of previous studies and explore the potential for distinct aetiologies of each autism-associated trait, ii) examine nonlinear associations between vitamin D in pregnancy and the offspring outcomes using conventional observational analyses and iii) use MR to improve causal inference of any linear associations.

## Methods

### Participants

**Fig. 1:**
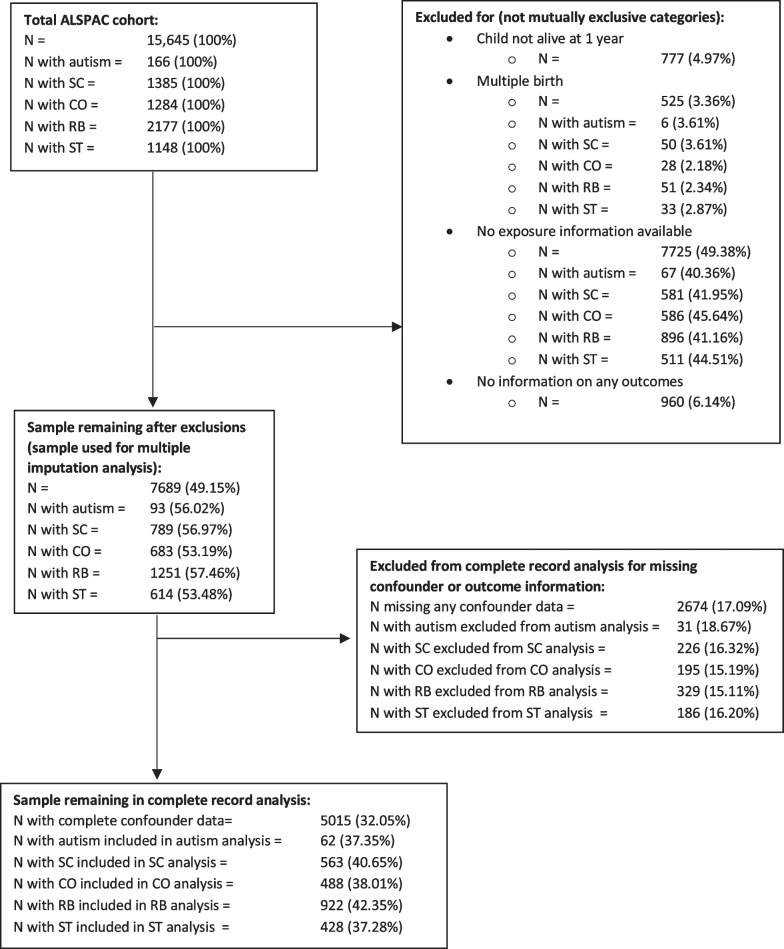
Flow chart of cohort derivation. Legend: SC = social communication trait; CO = speech coherence trait; RB = repetitive behaviour trait; ST = sociability temperament trait; AFM = autism factor mean score. Denominators for percentages taken from total ALSPAC cohort (top left-hand box)

Data were taken from ALSPAC [28, 29] which recruited 14,541 pregnant women residents in Avon, UK, with expected dates of delivery from 1 April 1991 to 31 December 1992. Eligibility criteria for this study were being a singleton pregnancy, surviving to one year, and not having withdrawn consent by the time of analysis. Mother and singleton offspring pairs were eligible if the mother had at least one valid measure of maternal 25(OH)D concentrations in pregnancy and the offspring had at least one outcome measure available. This left a total sample size of 7689 (see Fig. 1 for a flow chart of exclusions).

MR analyses were restricted to individuals of European ancestry to prevent bias from population stratification [30]. To achieve this, we restricted the main sample to individuals with genetic information available who were of European ancestry as detected by a multidimensional scaling analysis seeded with HapMap 2 individuals [31, 32]. This left a sample of 4718 individuals of European ancestry.

Please note the ALSPAC study website contains details of all the data that are available through a fully searchable data dictionary (http://www.bristol.ac.uk/alspac/researchers/our-data/).

### Exposure—maternal vitamin D during pregnancy

Maternal circulating total 25(OH)D (measured in nmol/L) in pregnancy, adjusted for gestational age and season at measurement, was used as the exposure. Serum samples could be from any stage of pregnancy. If a woman had more than one result, we used the latest. The procedure for sample extraction, storage, measurement and derivation of the adjusted variable has been described previously [33]. Briefly, our exposure was derived by log transforming the measure of maternal 25(OH)D, modelling this value according to the date of the blood sampling using linear regression with trigonometric sine and cosine functions, and then taking the residuals from this model. The model and its residuals were then used to adjust the total serum 25(OH)D to a pre-specified gestational age for each mother. A measure was derived for the midpoint of each trimester (adjusted to 7 weeks for trimester one, 20 weeks for trimester two and 34 weeks for trimester three). We further derived categorical variables for the exposure adjusted for season and to 20 weeks gestation, classifying < 25 nmol/L as deficient, 25–50 nmol/L as insufficient and > 50 nmol/L as sufficient.

### Outcomes—autism diagnosis and autism-associated traits

Children with autism have previously been identified in the ALSPAC cohort using a multi-source approach [34, 35]. This approach included a review of clinical records of all children who had a multidisciplinary assessment for a developmental disorder (validated against International Statistical Classification of Diseases, 10th Revision [ICD-10] criteria by a consultant paediatrician [36]), educational records of special education support provided for autism, and parental reports of an autism or Asperger syndrome diagnosis [34]. The autism cases have been cross-validated against autism-associated trait measures [34, 37], and the variable is associated with a genetic risk score for autism derived from an independent GWAS [35].

A measure of the broad autism phenotype, the autism factor mean score, has also been derived in ALSPAC as the mean of seven factors predictive of an autism diagnosis, produced by factor analysis [38]. This normally distributed measure was inverted so that higher values of the score reflected more autism-associated difficulties and was then standardised.

Four separate autism-associated trait measures, identified by Steer and colleagues [38] as being the best of 93 trait measures to be independent predictors of autism diagnosed using ICD-10 criteria, were also used as outcomes. These traits consisted of: the social communication difficulties trait—assessed at age 7 years via the Social Communication Disorder Checklist (SCDC) [39]; the speech coherence trait—assessed at age 9 years via a subscale of the Children’s Communication Checklist [40]; the repetitive behaviour trait—assessed at age 5 years via parental questionnaire response; and the sociability temperament trait—low sociability was assessed at age 3 years via a subscale of the Emotionality, Activity and Sociability Temperament Scale [41]. Binary measures were derived to approximately reflect the top 15% of scores on each trait, as has been described in detail previously [34].

### Potential confounders

Potential confounders adjusted for in-analysis models were selected a priori based on associations with both exposure and outcome in the literature. These included parity (0, 1, 2+), maternal age, pre-pregnancy BMI, maternal reported smoking status at 18 weeks gestation (yes/no) and measures of socio-economic status. The measures of socio-economic status were financial difficulties during pregnancy (yes/no), maternal highest education level (vocational/pre-age 16 schooling/post-age 16 schooling) and maternal occupational class (manual/non-manual). Further details on the selection and derivation of confounders are presented in the additional file, section 1.1.

In addition to these confounders, we also adjusted for offspring biological sex at birth (male/female) as there are reported strong sex differences in autism risk in childhood [42] and this adjustment could improve the statistical efficiency of our analyses.

We further considered maternal ethnicity to be a confounder due to its association with both vitamin D levels and risk of autism diagnosis [43]. Based on self-report, 87.9% of the sample mothers were White and 10.1% had missing ethnicity. Adjusting for this variable would lead to low or zero counts of the outcome in several strata. To account for the potential for confounding by ethnicity, we therefore repeated all primary analyses using the sample restricted to individuals with European ancestry as used in MR analyses.

### Statistical analyses

Analyses were performed in Stata version 17. We created quintiles of maternal 25(OH)D levels (adjusted to 20 weeks gestation) and assessed the prevalence of categorical covariates and the mean and standard deviation of continuous covariates across these quintiles.

### Missing data assessment

To assess associations with missing data and assess the likelihood of bias we compared the prevalence/means of exposure, outcome, confounders and auxiliary variables between those included in the sample and those excluded. We further performed logistic regression of being included in complete record analysis on each variable without adjustment. Complete record analysis has been shown to be biased when the probability of missing data is jointly dependent on both the exposure and the outcome for logistic regression while linear regression is biased when the probability of missing data is dependent on the outcome alone [44].

### Primary analysis

We performed regression models of outcomes on seasonal and gestational age-adjusted maternal 25(OH)D as a continuous variable using complete records only. All analyses were repeated for each of the outcomes and for each exposure measure, adjusted to different weeks of gestation. Linear regression was used where the outcome was continuous (autism factor mean score) and logistic regression was used for all binary outcomes (all remaining outcomes). For each outcome, we ran models (i) unadjusted, (ii) adjusted for offspring sex, (iii) adjusted for financial difficulties, maternal education and maternal occupational class, (iv) adjusted for parity, maternal age at birth, pre-pregnancy BMI and smoking status during pregnancy and (v) adjusted for all potential confounders. The exposure was entered into the model such that the output reflects the odds ratio/mean difference in outcome per 10 nmol/L higher seasonal and gestational age-adjusted 25(OH)D. Analyses were repeated using the categorical version of the exposure variable.

To account for potential bias from missing data, we performed multiple imputation [45, 46] with chained equations [47] and 100 imputations. All imputed data sets were created as part of the same procedure that included all outcomes, exposures and confounders in each prediction model. Auxiliary variables that were predictive of missing values and the probability of having a missing value were also included in all prediction models in order to meet the missing at random assumption required by standard multiple imputation implementation. This assumption states that the probability of missing data is not dependent on unobserved information, conditional on the observed information. These auxiliary variables included homeownership and marital status, while each outcome acted as an auxiliary variable for all other outcomes. Details of these auxiliary variables can be found in the additional file, section 1.2.

### Secondary analyses

To investigate a potential nonlinear association between outcomes and 25(OH)D we used restricted cubic regression splines with five knots in regression models adjusted for all potential confounders. This was repeated for each outcome (logistic regression for binary outcomes and linear regression for continuous outcomes) for the measure of 25(OH)D adjusted to 20-week gestation only. A reference value equal to the median of the adjusted 25(OH)D distribution (58.7 nmol/L) was used. As the number of knots is defined by the user and guidance on cubic regression splines states that 5 or fewer knots is normally appropriate [48], we tested the sensitivity of our conclusions from the spline analyses by altering the number of knots used.

### Mendelian randomization

We used one-sample MR [49–51] to explore evidence for a linear effect of gestational 25(OH)D on offspring autism diagnosis and associated traits. MR uses genotypes as instrumental variables under three core assumptions: (1) the instrument is robustly related and relevant to the exposure (the relevance assumption), (2) there is no confounding between the instrument and outcome (the independence assumption) and (3) the instrument is not associated with the outcome except through its association with the exposure (the exclusion restriction criteria).

This analysis used a standardised maternal genetic risk score (GRS) for higher 25(OH)D levels as an instrument to estimate possible causal effects on offspring outcomes. Genetic variants included in this GRS were selected from the latest genome-wide association study (GWAS) of serum 25(OH)D concentrations which included 401,460 White British men and women [52]. The GWAS identified 138 conditionally independent single nucleotide polymorphisms (SNP) from 69 distinct loci that were genome-wide significant (*p* < 5×10^–8^). We removed 2 palindromic SNPs with effect allele frequency between 0.4 and 0.6 to prevent the error from potentially using the wrong DNA strand. Of the remaining 136 SNPs, 72 were available in ALSPAC with minor allele frequency greater than 0.01 (see Additional file 1: Table S1). The GRS was then created as the weighted sum of the number of alleles an individual had (range 0–2) with weights based on the GWAS summary statistics, equal to the effect estimate divided by its standard error. The score was then standardised to mean 0 and variance 1. Further details of genotype information and derivation of the GRS are provided in the additional file section 1.3.

For the autism factor mean score, we used two-stage least squares regression (implemented using Stata’s *ivregress* command) to produce an estimate of the causal mean difference in outcome per 10 nmol/L increase in adjusted maternal 25(OH)D. For all binary outcomes, we used the logistic two-stage residual inclusion method (described elsewhere as the control function estimator) [53] to produce an estimate of the causal odds ratio per 10 nmol/L increase in adjusted maternal 25(OH)D. This was estimated using the *ivtsri* command from the *ivonesamplemr* Stata package [54].

To explore the relevance of the GWAS-derived genetic instrument with 25(OH)D in pregnancy we compared the association of the GRS with 25(OH)D in pregnancy in ALSPAC with the equivalent result from the GWAS. The F-statistic and *R*^2^ from a regression model of maternal 25(OH)D (adjusted to 20-week gestation) against maternal GRS were used as indicators of instrument strength. Confounding of the genetic instrument–outcome association can occur in the presence of population stratification. We attempted to make the independence assumption more plausible by restricting all MR analyses to mothers with European ancestry, and we further adjusted all analyses for 10 maternal genetic principal components. The association of maternal genotype with offspring genotype could violate the exclusion restriction criteria. We therefore repeated analyses adjusting for child GRS for 25(OH)D. To explore the presence of horizontal pleiotropy, we investigated the association between the maternal GRS and all risk factors for the outcomes and planned to do multivariable MR to adjust for the effect of any factors associated with it. A priori we decided not to explore nonlinear effects using MR as we did not feel we would have sufficient power.

## Results

Descriptive statistics for the sample are presented in Table 1, separated by quintiles of maternal adjusted 25(OH)D. The table shows that nulliparous mothers, mothers who smoked and had manual jobs, were less likely to be in the higher quintiles, while older mothers and those with higher education were more likely to be in the higher quintiles. There was no clear link between maternal adjusted 25(OH)D in pregnancy and offspring sex, BMI or financial difficulties.

**Table 1:** Descriptive statistics for quintiles of seasonally and gestational age (20 weeks) adjusted maternal serum 25(OH)D

	Total	Quintile of maternal serum 25(OH)D adjusted for season and to 20-week gestation					*p* value ^a^
		1	2	3	4	5	
	*N* = 7689	*N* = 1538	*N* = 1538	*N* = 1538	*N* = 1538	*N* = 1537	
**Covariates**							
Offspring male sex	4001 (52.0%)	791 (51.4%)	819 (53.3%)	816 (53.1%)	799 (52.0%)	776 (50.5%)	0.52
Parity							< 0.001
0	3242 (42.2%)	713 (46.4%)	677 (44.0%)	631 (41.0%)	607 (39.5%)	614 (39.9%)	
1	2527 (32.9%)	458 (29.8%)	498 (32.4%)	511 (33.2%)	531 (34.5%)	529 (34.4%)	
2 +	1430 (18.6%)	247 (16.1%)	277 (18.0%)	293 (19.1%)	304 (19.8%)	309 (20.1%)	
Missing	490 (6.4%)	120 (7.8%)	86 (5.6%)	103 (6.7%)	96 (6.2%)	85 (5.5%)	
Any maternal smoking at 18-week gestation	1935 (26.4%)	481 (33.2%)	432 (29.4%)	366 (25.2%)	330 (22.5%)	326 (22.0%)	< 0.001
Maternal highest educational qualification							0.024
Vocational	702 (9.1%)	131 (8.5%)	149 (9.7%)	147 (9.6%)	137 (8.9%)	138 (9.0%)	
CSE/O level	3824 (49.7%)	791 (51.4%)	773 (50.3%)	755 (49.1%)	778 (50.6%)	727 (47.3%)	
A level/degree	2435 (31.7%)	439 (28.5%)	462 (30.0%)	496 (32.2%)	489 (31.8%)	549 (35.7%)	
Missing	728 (9.5%)	177 (11.5%)	154 (10.0%)	140 (9.1%)	134 (8.7%)	123 (8.0%)	
Financial difficulties in pregnancy	498 (7.3%)	94 (7.0%)	101 (7.4%)	112 (8.2%)	94 (6.8%)	97 (7.0%)	0.66
Maternal manual occupation	1104 (19.5%)	223 (20.7%)	242 (21.7%)	210 (18.2%)	212 (18.5%)	217 (18.4%)	0.12
Maternal age at delivery	28.1 (4.8)	27.3 (5.0)	27.6 (4.9)	28.2 (4.9)	28.4 (4.7)	28.8 (4.7)	< 0.001
Maternal pre-pregnancy BMI	22.9 (3.8)	22.9 (4.0)	23.0 (4.0)	23.1 (3.9)	22.8 (3.6)	22.7 (3.6)	0.13
**Exposure**							
Maternal 25(OH)D (nMol/L) adjusted to 7 weeks ^b^	64.1 (32.0)	34.8 (10.9)	50.6 (14.3)	61.5 (18.6)	73.4 (23.4)	100.3 (38.4)	< 0.001
Maternal 25(OH)D (nMol/L) adjusted to 20 weeks ^b^	64.6 (31.7)	29.3 (6.4)	45.2 (4.0)	58.9 (4.2)	75.6 (5.9)	114.1 (26.1)	< 0.001
Maternal 25(OH)D (nMol/L) adjusted to 34 weeks ^b^	69.8 (34.1)	37.3 (12.5)	55.0 (16.7)	67.9 (22.2)	81.7 (27.6)	107.0 (36.3)	< 0.001
Maternal Standardised Genetic 25(OH)D score ^b^	−0.0 (1.0)	−0.1 (1.0)	−0.0 (1.0)	−0.1 (1.0)	0.1 (1.0)	0.1 (1.0)	< 0.001
Offspring Standardised Genetic 25(OH)D score ^b^	0.0 (1.0)	−0.1 (1.0)	−0.1 (1.0)	0.0 (1.0)	0.1 (1.0)	0.1 (1.0)	0.010
**Outcome**							
Diagnosed Autism	93 (1.2%)	18 (1.2%)	17 (1.1%)	22 (1.4%)	23 (1.5%)	13 (0.8%)	0.47
Social Communication Trait	789 (17.0%)	159 (18.9%)	156 (17.0%)	164 (17.0%)	155 (16.4%)	155 (16.1%)	0.58
Speech Coherence Trait	683 (15.5%)	136 (17.4%)	140 (16.1%)	138 (15.0%)	122 (13.4%)	147 (15.7%)	0.21
Repetitive Behaviour Trait	1251 (27.6%)	258 (30.7%)	247 (27.9%)	236 (25.6%)	268 (28.1%)	242 (26.2%)	0.14
Sociability Temperament Trait	614 (10.8%)	125 (11.5%)	113 (10.2%)	139 (12.0%)	114 (9.7%)	123 (10.6%)	0.37
Autism factor mean score ^b^	−0.0 (1.0)	0.1 (1.1)	0.0 (1.0)	−0.0 (0.9)	−0.0 (1.0)	−0.0 (1.0)	0.039
**Auxiliary variables**							
Home ownership status							< 0.001
Owned/mortgaged	5406 (70.3%)	987 (64.2%)	1050 (68.3%)	1110 (72.2%)	1113 (72.4%)	1146 (74.6%)	
Council rented	1057 (13.7%)	255 (16.6%)	228 (14.8%)	206 (13.4%)	202 (13.1%)	166 (10.8%)	
Privately rented	565 (7.3%)	138 (9.0%)	141 (9.2%)	88 (5.7%)	98 (6.4%)	100 (6.5%)	
Other	248 (3.2%)	56 (3.6%)	48 (3.1%)	59 (3.8%)	41 (2.7%)	44 (2.9%)	
Missing	413 (5.4%)	102 (6.6%)	71 (4.6%)	75 (4.9%)	84 (5.5%)	81 (5.3%)	
Marital status							< 0.001
Never married	1359 (17.7%)	334 (21.7%)	316 (20.5%)	252 (16.4%)	231 (15.0%)	226 (14.7%)	
Previously married (currently unmarried)	429 (5.6%)	78 (5.1%)	96 (6.2%)	100 (6.5%)	72 (4.7%)	83 (5.4%)	
1st marriage	5033 (65.5%)	940 (61.1%)	961 (62.5%)	1008 (65.5%)	1077 (70.0%)	1047 (68.1%)	
2nd or 3rd marriage	475 (6.2%)	83 (5.4%)	93 (6.0%)	101 (6.6%)	82 (5.3%)	116 (7.5%)	
Missing	393 (5.1%)	103 (6.7%)	72 (4.7%)	77 (5.0%)	76 (4.9%)	65 (4.2%)	

^a^*p* values produced using ANOVA for continuous variables and Pearson’s chi-squared test for binary and categorical variables

^b^Mean (SD)

We provide a plot of 25(OH)D against the date of measurement in Additional file 1: Figure S1 (additional file section 2.1) to illustrate the seasonal adjustment strategy. A histogram of adjusted maternal 25(OH)D can be seen in Additional file 1: Figure S2. Maternal 25(OH)D adjusted to 20-week gestation was below 25 nmol/L (deficient) for 5.0% of the sample, between 25 and 50 nmol/L (insufficient) for 32.1%, and greater than 50 nmol/L (sufficient) for 62.8%.

Additional file 1: Table S2 (additional file section 2.2) shows descriptive statistics for those who were excluded vs included based on a priori inclusion/exclusion criteria. Mothers who smoked had financial difficulties and worked manual jobs were less likely to be included, while those who were older were more likely to be included. Higher maternal 25(OH)D adjusted to each time point was associated with increased odds of inclusion. Children with difficulties in social communication, speech coherence and sociability traits as well as higher autism factor mean scores were associated with lower odds of inclusion. The GRS for 25(OH)D was not associated with odds of inclusion.

### Missing data assessment

Descriptive statistics for the inclusion in complete record analyses are presented in Additional file 1: Tables S3 and S4 (additional file section 2.3). A 10 nmol/L increase in exposure was associated with a small increase in the odds of having a complete record for all outcomes. Autism diagnosis was not associated with having a complete record. Case status for all autism-associated traits and higher autism factor mean score were associated with reduced odds of having a complete record, meaning that bias in complete record analyses for these outcomes is likely. The GRS for 25(OH)D was not associated with having a complete record. Regarding the auxiliary variables, home ownership and marital status both strongly predicted the probability of having a complete record. Those who did not own their own home were much less likely to have complete records, while those who were currently married were more likely to have complete records.

### Primary analysis

The results of the primary analysis are presented in Table 2 for all binary outcomes. There was no strong evidence for an association between any of the binary outcomes and maternal 25(OH)D adjusted to 20-week gestation in either unadjusted or adjusted multivariable regression models using complete record analysis or multiple imputation. Table 3 presents the results for the autism factor mean score. There was evidence for an association between higher 25(OH)D levels adjusted to 20-week gestation and a lower autism factor mean score in the unadjusted multiple imputation model; however, the effect estimate was small.

**Table 2:** Logistic regression of each outcome on maternal 25(OH)D adjusted for season and 20-week gestation

Outcome	Model	Complete record analysis		Multiple imputation analysis	
		OR	95% CI	OR	95% CI
Autism diagnosis	Unadjusted	0.99	(0.91, 1.07)	1.00	(0.94, 1.07)
	Adjusted 1	0.99	(0.91, 1.07)	1.01	(0.94, 1.07)
	Adjusted 2	0.99	(0.91, 1.07)	1.00	(0.94, 1.07)
	Adjusted 3	0.98	(0.90, 1.06)	0.99	(0.93, 1.06)
	Fully adjusted	0.98	(0.90, 1.06)	0.99	(0.93, 1.06)
Social communication trait	Unadjusted	1.00	(0.97, 1.03)	0.99	(0.97, 1.02)
	Adjusted 1	1.00	(0.97, 1.02)	0.99	(0.97, 1.02)
	Adjusted 2	1.00	(0.97, 1.03)	0.99	(0.97, 1.02)
	Adjusted 3	1.00	(0.97, 1.03)	1.00	(0.98, 1.03)
	Fully adjusted	1.00	(0.97, 1.03)	1.00	(0.98, 1.03)
Speech coherence trait	Unadjusted	0.99	(0.96, 1.02)	1.00	(0.98, 1.02)
	Adjusted 1	0.99	(0.96, 1.02)	1.00	(0.98, 1.02)
	Adjusted 2	0.99	(0.96, 1.02)	1.00	(0.98, 1.03)
	Adjusted 3	0.99	(0.96, 1.02)	1.00	(0.98, 1.03)
	Fully adjusted	0.99	(0.96, 1.02)	1.00	(0.98, 1.03)
Repetitive behaviour trait	Unadjusted	0.99	(0.96, 1.01)	0.98	(0.96, 1.00)
	Adjusted 1	0.98	(0.96, 1.01)	0.98	(0.96, 1.00)
	Adjusted 2	0.99	(0.96, 1.01)	0.98	(0.96, 1.00)
	Adjusted 3	0.99	(0.96, 1.01)	0.98	(0.96, 1.00)
	Fully adjusted	0.99	(0.96, 1.01)	0.98	(0.96, 1.00)
Sociability temperament trait	Unadjusted	1.01	(0.97, 1.04)	0.99	(0.97, 1.02)
	Adjusted 1	1.00	(0.97, 1.04)	1.00	(0.97, 1.02)
	Adjusted 2	1.01	(0.98, 1.04)	1.00	(0.97, 1.02)
	Adjusted 3	1.00	(0.97, 1.04)	1.00	(0.97, 1.02)
	Fully adjusted	1.00	(0.97, 1.04)	1.00	(0.97, 1.02)

Odds ratios reflect a 10 nmol/L change in adjusted 25(OH)D

Adjusted 1 = adjusted for offspring sex;

Adjusted 2 = adjusted for financial difficulties, maternal education, and maternal occupational class;

Adjusted 3 = adjusted for parity, maternal age at birth, pre-pregnancy BMI and smoking status during pregnancy;

Fully adjusted = adjusted for all variables in adjusted 1–3

Complete record analysis N = 5013 for autism diagnosis; 3526 for social communication trait; 3394 for speech coherence trait; 3464 for repetitive behaviour trait; 4209 for sociability temperament trait

Multiple imputation analysis N = 7689 for all outcomes

**Table 3:** Autism factor mean score regression on maternal 25(OH)D adjusted for season and 20-week gestation

Analysis	Model	Complete record analysis		Multiple imputation analysis	
		Mean difference*	95% CI	Mean difference	95% CI
Observational^a^	Unadjusted	−0.01	(−0.01, 0.00)	−0.01	(−0.02, −0.00)
	Adjusted 1	−0.01	(−0.01, 0.00)	−0.01	(−0.01, 0.00)
	Adjusted 2	0.00	(−0.01, 0.00)	−0.01	(−0.01, 0.00)
	Adjusted 3	−0.01	(−0.01, 0.00)	−0.01	(−0.01, 0.00)
	Fully adjusted	−0.01	(−0.01, 0.00)	−0.01	(−0.01, 0.00)
Mendelian randomization^b^	Adjusted for 10 principal components	−0.04	(−0.13, 0.04)	–	–
	Adjusted for 10 principal components and offspring risk score for 25(OH)D	−0.05	(−0.20, 0.09)	–	–

^a^Mean difference in autism factor mean score per 10 nmol/L change in adjusted 25(OH)D

^b^Mean difference in autism factor mean score per 10 nmol/L change in adjusted 25(OH)D per unit increase in standardised maternal 25(OH)D genetic score

^*^Causal mean difference for Mendelian randomization

Adjusted 1 = adjusted for offspring sex;

Adjusted 2 = adjusted for financial difficulties, maternal education, and maternal occupational class;

Adjusted 3 = adjusted for parity, maternal age at birth, pre-pregnancy BMI and smoking status during pregnancy;

Fully adjusted = adjusted for all variables in adjusted 1–3

Complete record analysis N = 4913; multiple imputation analysis N = 7689

Mendelian randomization analysis, unadjusted N = 4462, adjusted N = 2937

Primary analyses using maternal 25(OH)D adjusted to 7-week gestation and 34-week gestation did not differ substantially from the results adjusted to 20-week gestation (see Additional file 1: Table S5 and S6; additional file section 2.4). Similarly, results from analyses repeated using a sample with European ancestry only did not differ meaningfully from those in the full sample (Additional file 1: Table S7 in section 2.5). Analyses repeated using a categorical measure of the exposure adjusted to 20-week gestation are displayed in Table 4. We found some evidence of increased odds of the speech coherence trait and the repetitive behaviour trait among offspring of mothers with insufficient as compared to sufficient 25(OH)D levels in complete record but not multiple imputation analyses. No other associations were suggested.

**Table 4:** Primary analyses repeated using categorical measures of maternal circulating total 25(OH)D (nmol/L) in pregnancy

Outcome	Vitamin D group	Complete record analysis		Multiple imputation analysis	
		Effect estimate	95% CI	Effect estimate	95% CI
Autism diagnosis ^a^	**Unadjusted**				
	Deficient	0.76	(0.18, 3.17)	0.93	(0.37, 2.33)
	Insufficient	1.20	(0.71, 2.04)	0.94	(0.36, 2.43)
	**Fully adjusted**				
	Deficient	0.91	(0.22, 3.83)	0.79	(0.31, 1.99)
	Insufficient	1.28	(0.75, 2.19)	0.85	(0.33, 2.22)
Social communication trait ^a^	**Unadjusted**				
	Deficient	1.18	(0.76, 1.82)	0.93	(0.66, 1.32)
	Insufficient	1.11	(0.91, 1.35)	1.01	(0.71, 1.42)
	**Fully adjusted**				
	Deficient	1.14	(0.73, 1.78)	1.00	(0.70, 1.41)
	Insufficient	1.08	(0.88, 1.31)	1.02	(0.72, 1.45)
Speech coherence trait ^a^	**Unadjusted**				
	Deficient	1.15	(0.71, 1.88)	0.95	(0.67, 1.34)
	Insufficient	1.31	(1.06, 1.60)	1.07	(0.76, 1.51)
	**Fully adjusted**				
	Deficient	1.17	(0.71, 1.92)	0.96	(0.68, 1.36)
	Insufficient	1.31	(1.06, 1.61)	1.07	(0.75, 1.51)
Repetitive behaviours trait^a^	**Unadjusted**				
	Deficient	1.32	(0.92, 1.91)	0.83	(0.64, 1.09)
	Insufficient	1.18	(1.00, 1.39)	0.97	(0.74, 1.28)
	**Fully adjusted**				
	Deficient	1.30	(0.90, 1.88)	0.86	(0.66, 1.13)
	Insufficient	1.17	(0.99, 1.38)	0.97	(0.74, 1.28)
Sociability temperament trait^a^	**Unadjusted**				
	Deficient	0.60	(0.32, 1.11)	1.14	(0.75, 1.72)
	Insufficient	1.12	(0.91, 1.39)	1.23	(0.80, 1.87)
	**Fully adjusted**				
	Deficient	0.61	(0.32, 1.14)	1.14	(0.75, 1.72)
	Insufficient	1.15	(0.93, 1.43)	1.23	(0.80, 1.88)
Autism factor mean score^b^	**Unadjusted**				
	Deficient	0.03	(−0.10, 0.16)	−0.08	(−0.19, 0.03)
	Insufficient	0.10	(0.04, 0.15)	0.00	(−0.12, 0.11)
	**Fully adjusted**				
	Deficient	0.05	(−0.08, 0.17)	−0.09	(−0.19, 0.02)
	Insufficient	0.09	(0.04, 0.15)	−0.02	(−0.13, 0.09)

Deficient: < 25 nmol/L; insufficient: 25–50 nmol/L; sufficient: > 50 nmol/L

All estimates relative to sufficient vitamin D

Fully adjusted = adjusted for offspring sex, financial difficulties, maternal education, and maternal occupational class, parity, maternal age at birth, pre-pregnancy BMI and smoking status during pregnancy

^a^Effect estimate = odds ratio

^b^Effect estimate = mean difference

### Secondary analysis

**Fig. 2:**
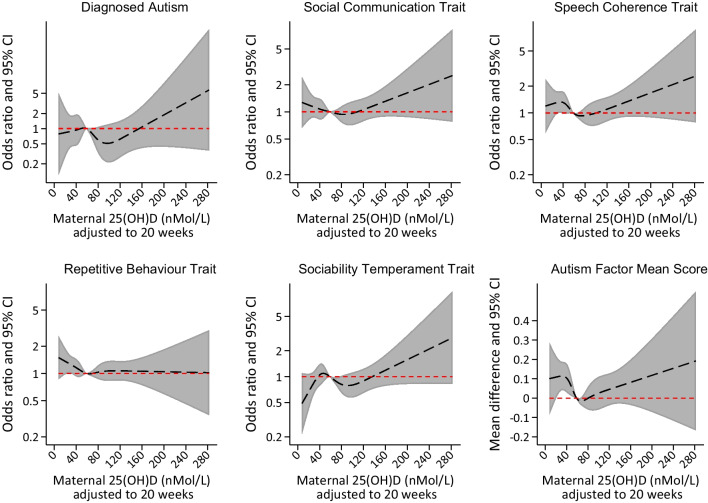
Regression models of each outcome on adjusted 25(OH)D using restricted cubic regression splines (five knots). Legend: model adjusted for parity, offspring sex, maternal age at birth, pre-pregnancy BMI, smoking status during pregnancy, financial difficulties during pregnancy, maternal education and maternal occupational class

Figure 2 shows the results of the spline analysis. There was some suggestion of increased odds of meeting case criteria on the social communication and speech coherence measures and having a higher score on the mean autism factor measure with values of maternal 25(OH)D that were lower or higher than the median for the sample. Strong evidence was only found for the autism factor mean score, and only for lower levels of maternal 25(OH)D. We considered the possibility that this was an artefact related to splitting the data into fifths and having limited power within each fifth. Sensitivity analyses that split the data into a smaller number of groups (quarters or thirds) provided stronger evidence for associations between low, but not high, levels of maternal 25(OH)D and higher odds of offspring speech coherence and higher autism factor mean scores when the data were split into thirds (see Additional file 1: Figures S3 and S4 in section 2.6).

### Mendelian randomization analysis

The maternal GRS predicted 1.05% of the variance in maternal 25(OH)D scores adjusted to 20-week gestation (mean difference in 25(OH)D per unit change in the risk score = 3.24 nmol/L; 95% CI = 2.34–4.14) and had an F-statistic of 50.26. This is less than the 3.2% of variance explained by common variants found by Manousaki et al. [52] suggesting the SNPs are a weaker predictor of 25(OH)D in women and/or during pregnancy than in non-pregnant women and men. The maternal GRS was not associated with any of the measured risk factors for the outcome providing evidence against the presence of horizontal pleiotropy (see Additional file 1: Table S8 in section 2.7). We therefore did not undertake multivariable MR.

**Fig. 3:**
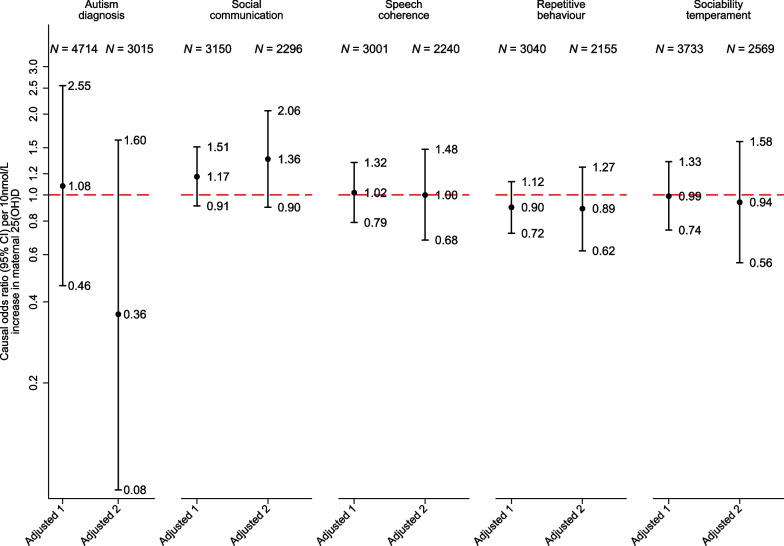
One-sample Mendelian randomization analyses of binary outcomes. Legend: adjusted 1 = adjusted for 10 principal components; adjusted 2 = adjusted for 10 principal components and for offspring genetic risk score. CI = Confidence interval

Results of the MR analyses are presented in Fig. 3 for all binary outcomes and Table 3 for the autism factor mean score. The MR analyses provided estimates further from the null but with less precision than the observational estimates. There was no strong evidence for an effect of 25(OH)D during pregnancy on any of the offspring outcomes. The conclusions from these analyses were not substantially changed by adjusting for the offspring GRS for 25(OH)D.

## Discussion

The results of the current study did not provide evidence for an association between maternal circulating 25(OH)D during pregnancy and autism diagnosis or any autism-associated trait. There was no strong evidence of an association when the exposure was entered as a continuous measure or categorised into levels of sufficiency once missing data had been accounted for. There was some evidence of increased odds of the social communication trait and speech coherence trait and evidence of an increased autism factor mean score using spline models; however, these analyses did not account for missing data. The MR analyses did not provide any evidence for a causal effect of maternal genetically proxied levels of 25(OH)D on offspring autism outcomes, though power was limited.

The results estimated in the ALSPAC cohort conflict with findings of meta-analyses of previous observational studies which suggested an increased risk of autism diagnoses and autism-associated traits among offspring of mothers with lower levels of 25(OH)D during pregnancy [3, 4]. The discrepancy is unlikely to be due to different timings of exposure measurement between studies because results in the current study were consistent between analyses that used measures of 25(OH)D adjusted to the predicted value at three separate time points of gestation. The difference is also unlikely to be the result of using a continuous measure of the exposure versus categorising as no evidence was found for an association using either form of the exposure once missing data had been accounted for.

ALSPAC had fewer participants classified as having deficient levels of 25(OH)D during pregnancy (5% predicted to be deficient at 20-week gestation) than some other studies, such as Generation R where 16% of mothers were deficient at 21-week gestation [12] and the Stockholm Youth Cohort where 8.6% of mothers were predicted to be deficient at 11-week gestation [13]. ALSPAC may therefore not adequately capture the extreme lows of the distribution of 25(OH)D, potentially as a result of the disproportionately affluent and White European population contained within the cohort as compared to the rest of the UK population [29].

Strengths of the current study include the use of multiple methods to assess the research question applied to the same data set. To our knowledge, this is the first time that MR has been applied to examine the association between 25(OH)D levels in pregnancy and offspring autism. Future work employing MR with larger sample sizes could help with triangulation as to whether a causal effect is likely to exist. Further, we were able to validate that our genetic score, derived from a general population GWAS, was predictive of 25(OH)D levels during pregnancy. Our use of seasonal and gestational age-adjusted measures of 25(OH)D helped to account for differences in the timing of exposure measurement, thereby removing this as a source of bias.

### Limitations

Our study had a relatively small sample size which may have resulted in low power to detect effects, particularly for all analyses involving diagnosed autism as an outcome. While counts for diagnosed cases of autism were low in our sample, they were representative of the prevalence in the UK population [55]. The power to detect a 1.5-fold increase in odds of autism diagnoses with the full sample (ignoring missing data in any variable) at the 0.05 alpha level was 53% for insufficient vs sufficient 25(OH)D levels and 22% for deficient vs sufficient levels. The power for all other binary outcomes was greater than 99% for insufficient 25(OH)D and ranged from 66 to 86% for deficient 25(OH)D.

Further limitations of the observational analyses include the possibility of residual confounding due to misreporting of smoking, which is common in pregnancy, and due to BMI being calculated using a maternal retrospective report of pre-pregnancy weight and height. There was potential for bias from missing data where the outcome and exposure were predictive of the probability of being excluded from complete record analyses [44]. We used MI with auxiliary variables to make the missing at random assumption more plausible and reduce any potential bias from missing data [45, 46].

Power was also likely to be low for MR estimates and our instrument explained a low proportion of variance in maternal 25(OH)D scores, challenging the relevance assumption of MR analyses. This is an issue common to most MR analyses, where instruments often explain only a small proportion of the variance in the exposure. To make the assumptions of the MR analyses more plausible, we used the latest and largest GWAS of 25(OH)D levels, conducted sensitivity analyses to test the instrument strength (F-statistic > 10) and adjusted for principal components and offspring GRS for 25(OH)D.

Adjustment for child 25(OH)D GRS in MR analyses reduced the likelihood of violating the exclusion restriction criteria; however, it may also have induced collider bias and opened a pathway via paternal genetics. It has been shown that any bias from paternal genetics is likely to be minor [51]. Dynastic effects and assortative mating may also have confounded the GRS–outcome association [30]. Such effects can be addressed using within-family analyses; however, data on sibling exposure, outcome and genetics were not available in ALSPAC [56].

## Conclusion

We have not found any evidence for an association between maternal 25(OH)D during pregnancy and offspring risk of autism or autism-associated traits using the ALSPAC cohort. This suggests that we should treat the emerging evidence for an association with caution. Few previous studies have applied causal inference techniques to this question. Triangulation of findings from causal inference analyses will be an important step in establishing whether 25(OH)D during pregnancy has a causal role in the origins of autism.

## Supplementary Information

**Additional file 1**. Supplementary material.

## Data Availability

ALSPAC data access is through a system of managed open access. The steps below highlight how to apply for access to ALSPAC data: (1) Please read the ALSPAC access policy (PDF, 627kB) (http://www.bristol.ac.uk/media-library/sites/alspac/documents/researchers/data-access/ALSPAC_Access_Policy.pdf) which describes the process of accessing the data and samples in detail and outlines the costs associated with doing so. (2) You may also find it useful to browse our fully searchable research proposals database (https://proposals.epi.bristol.ac.uk/), which lists all research projects that have been approved since April 2011. (3) Please submit your research proposal (https://proposals.epi.bristol.ac.uk/) for consideration by the ALSPAC Executive Committee. You will receive a response within 10 working days to advise you whether your proposal has been approved. If you have any questions about accessing data, please email alspac-data@bristol.ac.uk. All scripts used to create data sets from the ALSPAC cohort and to run analyses can be obtained from https://github.com/pmadleydowd/VitaminD_and_Autism.
