# Supplementary material for: Maternal vitamin D during pregnancy and offspring autism and autism-associated traits: a prospective cohort study
Source: Mol Autism. 2022 Nov 12;13:44. doi: 10.1186/s13229-022-00523-4 (PMC9652971; doi:10.1186/s13229-022-00523-4)
Supplement: Supplementary file 1 — Additional file 1. Supplementary material. [file 13229_2022_523_MOESM1_ESM.docx]

Supplementary material

Maternal vitamin D during pregnancy and offspring autism and autism-associated traits: a prospective cohort study

**Contents**

[1 Supplementary Methods 2](#_Toc118126471)

[1.1 - Further details on confounder selection and derivation 2](#_Toc118126472)

[1.2 - Auxiliary variables included in the imputation model 2](#_Toc118126473)

[1.3 - Mendelian randomisation analyses 3](#_Toc118126474)

[1.3.1 - Genotype information 3](#_Toc118126475)

[1.3.2 - Creating the genetic scores for vitamin D 3](#_Toc118126476)

[2 Supplementary Results 7](#_Toc118126477)

[2.1 - Distribution of seasonally and gestational age adjusted maternal vitamin D 7](#_Toc118126478)

[2.2 - Comparison of included and excluded participants 8](#_Toc118126479)

[2.3 - Missing data assessment 10](#_Toc118126480)

[2.4 - Primary analyses repeated with adjustment to 7 weeks and 34 weeks gestation 14](#_Toc118126481)

[2.5 - Primary analyses restricted to individuals of European ancestry 16](#_Toc118126482)

[2.6 - Sensitivity of spline analyses to the number of knots used 17](#_Toc118126483)

[2.7 - MR Sensitivity analysis 19](#_Toc118126484)

[3 References 20](#_Toc118126485)

# Supplementary Methods

## Further details on confounder selection and derivation

Evidence in the literature has suggested that parity (1, 2), maternal age (2-4), pre-pregnancy BMI (1, 3, 5, 6), maternal smoking status (3, 7, 8) and socioeconomic position (3, 4, 9) are plausibly associated with both exposure and outcome and may therefore bias estimates of association. These variables were selected a priori to be included as confounders for adjustment in models. While smoking in pregnancy has been suggested not to causally influence the risk of autism, smoking can act as an additional proxy for socioeconomic position (10).

Pre-pregnancy BMI was measured using retrospective report of height and weight before pregnancy via questionnaire administered either during pregnancy or 4 months after delivery.

Smoking status at 18 weeks gestation was measured using maternal self-report and coded as yes if the mother responded that they 1) had ever been a smoker and did not report that they had stopped smoking, or 2) reported that the number of cigarettes smoked in the first three months of pregnancy or in the last two weeks was greater than 0. Otherwise, mothers were coded as non-smokers.

Financial difficulties were measured using a self-report questionnaire during pregnancy. A score was derived from the sum of responses indicating the level of difficulty in affording food, clothing, heating, rent or mortgage and “things you will need for the baby”. Those in the highest decile of the score were indicated as having financial difficulties.

Maternal highest education level was recorded via self report questionnaire at 32 weeks gestation. The variable was grouped as 1) vocational qualification, 2) CSE or O level (UK qualifications from formal education up to age 16) or 3) A level or degree (UK qualifications from post age 16 education).

Maternal occupational class was also recorded via self report questionnaire at 32 weeks gestation and grouped into manual or non-manual occupation according to the definitions provided by the 1991 British Office of Population Census Surveys job codes (10).

## Auxiliary variables included in the imputation model

The following auxiliary variables were included in the imputation model:

- Homeownership status (available for 94.6% of the sample)
- Maternal marital status (available for 94.9% of the sample)
- Each outcome acted as an auxiliary variable for all other outcomes

Homeownership was grouped as (i) owned/mortgaged, (ii) rented from the council, (iii) privately rented, or (iv) other. Marital status was grouped as (i) never married, (ii) previously married but not currently married (this included those who were widowed, separated, and divorced), (iii) those who were in their first marriage, and (iv) those who were in their second or third marriage. These variables were predictive of socioeconomic position variables (financial difficulties – 11.3% missing, maternal education – 9.5% missing, and maternal occupational class – 26.2% missing) and were predictive of inclusion as a complete record in analyses.

Information on an autism diagnosis was available for 99.9% of participants while a autism factor mean score was available for 92.2% of the sample.

## Mendelian randomisation analyses

### Genotype information

Data on maternal genetic information was extracted from the ALSPAC genetic database (11). Genotyping of ALSPAC mothers was performed using the Illumina human660W-quad at Centre National de Génotypage and genotypes were called using the Illumina GenomeStudio algorithm. Quality control was undertaken using PLINK (v1.07) on an initial set of 10,015 subjects and 557,124 directly genotyped SNPs. SNPs were removed if they had a proportion of missing data greater than 5%, had a Hardy-Weinberg-Equilibrium p-value lower than 10^-6^ or had a minor allele frequency less than 1%. Samples were excluded if they had greater than 5% missing data, had indeterminate X chromosome heterozygosity or had extreme autosomal heterozygosity. The quality control process is described in further detail elsewhere (12). SNP imputation was carried out against the 1000 Genome Project database (13). Quality control and SNP imputation were undertaken by the ALSPAC team before access was granted to the data.

### Creating the genetic scores for vitamin D

We obtained summary statistics from a recent GWAS of serum 25-hydroxyvitamin D concentrations (14) using data from 401,460 White British male and female UK Biobank participants (15). This GWAS also included a meta-analysis which combined results with a previous GWAS of 42,274 Europeans that included ALSPAC participants. We therefore decided to use the results restricted to only Biobank participants, (note that these results are very similar to the meta-analysed results). The GWAS log transformed and standardised the measure of vitamin D concentrations and adjusted for age, sex, season of measurement and vitamin D supplementation.

Maternal genetic risk scores (GRS) for vitamin D in the ALSPAC cohort were then derived using PLINK (16, 17) as a weighted sum of the number of alleles an individual had (range 0-2) for each independent SNP obtained from the GWAS. Weightings for a given SNP were based on the GWAS summary statistics and were equal to the effect estimate divided by its standard error. Where individuals were missing data for a SNP, a contribution proportional to the imputed allele frequency was added to the risk score. Polygenic scores were standardised prior to analysis. Table S1 shows the list of 72 SNPs included in the GRS with their effect allele and weighting.

Table S1: The 72 SNPs included in the GRS for vitamin D

| SNP | Effect Allele | Weighting |
| --- | --- | --- |
| rs1011468 | A | -6.751102 |
| rs10127775 | T | 5.929463 |
| rs10426 | A | 10.111826 |
| rs1047891 | A | -6.082800 |
| rs10500209 | C | -5.960317 |
| rs10793129 | A | 6.981523 |
| rs10818769 | G | -5.927211 |
| rs10832218 | C | -11.142370 |
| rs10859995 | C | -18.936684 |
| rs10887718 | T | -5.887800 |
| rs11127048 | A | 9.284369 |
| rs11264360 | A | 6.974488 |
| rs1149605 | C | 7.465875 |
| rs11723621 | G | -81.196228 |
| rs117913124 | A | -56.155660 |
| rs12317268 | G | -6.835737 |
| rs12803256 | G | 41.470564 |
| rs12997242 | A | -6.021919 |
| rs157595 | G | -6.900187 |
| rs17765311 | C | -7.655028 |
| rs1800588 | T | -12.171037 |
| rs1800775 | A | -8.434740 |
| rs1858889 | C | 6.015211 |
| rs186881826 | A | 17.125389 |
| rs1972994 | T | -8.182755 |
| rs2011425 | G | -11.807388 |
| rs2037511 | A | 6.205968 |
| rs2074735 | C | 6.605546 |
| rs222026 | T | -16.451582 |
| rs2229742 | C | -7.341194 |
| rs2585442 | G | 13.751983 |
| rs261291 | C | -11.190901 |
| rs28364331 | G | 8.101349 |
| rs2847500 | A | -7.180610 |
| rs2909218 | T | 6.628200 |
| rs34726834 | T | 5.730098 |
| rs3750296 | C | -9.681924 |
| rs3768013 | A | -6.468691 |
| rs523583 | C | 5.708922 |
| rs532436 | A | -5.793995 |
| rs56044892 | T | 5.182067 |
| rs57631352 | G | -5.616592 |
| rs58073039 | G | -6.691716 |
| rs58542926 | T | 8.624578 |
| rs6123359 | G | 9.363555 |
| rs6127099 | T | -14.647312 |
| rs61816761 | A | 17.950764 |
| rs62007299 | A | -6.257105 |
| rs6438900 | G | 6.003431 |
| rs6698680 | G | -5.616667 |
| rs6724965 | G | -5.629630 |
| rs6773343 | T | 5.345614 |
| rs705117 | T | -12.217468 |
| rs71383766 | T | 6.373233 |
| rs73015021 | G | 7.723057 |
| rs7519574 | A | 6.119228 |
| rs7528419 | G | 7.933498 |
| rs7569755 | A | 6.425011 |
| rs7699711 | T | -14.171260 |
| rs7718395 | G | 5.668787 |
| rs77924615 | A | -5.990705 |
| rs7828742 | G | -10.764959 |
| rs78649910 | A | -5.914907 |
| rs8018720 | C | -11.419850 |
| rs804280 | A | 6.856589 |
| rs8063706 | T | 6.012179 |
| rs8091117 | A | -5.917493 |
| rs8103262 | C | 5.218764 |
| rs867772 | G | -6.549566 |
| rs960596 | T | 5.653278 |
| rs964184 | C | 13.791611 |
| rs9668081 | T | 5.863770 |

# Supplementary Results

## Distribution of seasonally and gestational age adjusted maternal vitamin D


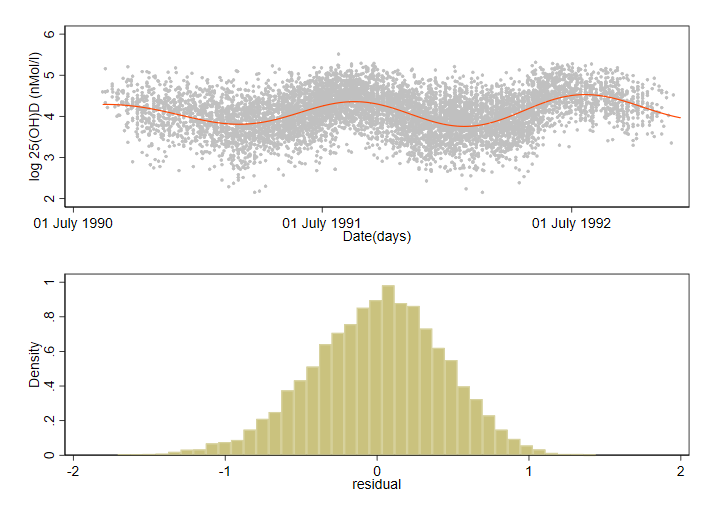


Figure S1: Plots of (top) log(25(OH)D against the date of measurement with a fitted line for the expected value for the calendar time of measurement and (bottom) a histogram of the residuals for the fitted line in the plot above.


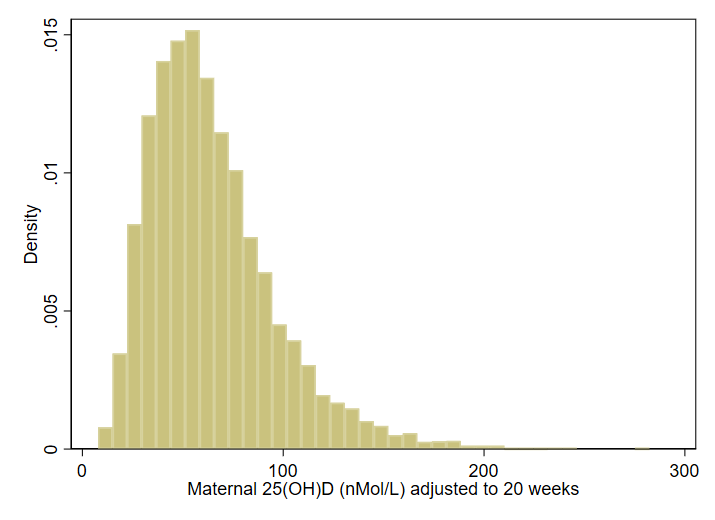


Figure S2: Histogram of maternal 25(OH)D adjusted for season and to 20 weeks gestation.

## Comparison of included and excluded participants

Table S2: Descriptives for participants excluded and included from analyses. Odds ratios (OR) reflect the odds of inclusion into the study sample.

|  | Total | Excluded from  analysis sample | Included in  analysis sample |  |
| --- | --- | --- | --- | --- |
| Variable | N=15,645 | N=7,956 | N=7,689 | OR (95% CI) for inclusion |
| Covariates |  |  |  |  |
| Offspring male sex | 7,690 (51.1%) | 3,689 (50.2%) | 4,001 (52.0%) | 1.07 (1.00-1.14) |
| Parity |  |  |  |  |
| 0 | 5,866 (37.5%) | 2,624 (33.0%) | 3,242 (42.2%) | Ref |
| 1 | 4,581 (29.3%) | 2,054 (25.8%) | 2,527 (32.9%) | 0.86 (0.79-0.93) |
| 2+ | 2,662 (17.0%) | 1,232 (15.5%) | 1,430 (18.6%) | 0.53 (0.48-0.58) |
| Missing | 2,536 (16.2%) | 2,046 (25.7%) | 490 (6.4%) |  |
| Any maternal smoking at 18 weeks gestation | 3,597 (27.0%) | 1,662 (27.6%) | 1,935 (26.4%) | 0.63 (0.58-0.69) |
| Maternal highest educational qualification |  |  |  |  |
| Vocational | 1,229 (7.9%) | 527 (6.6%) | 702 (9.1%) | Ref |
| CSE/O level | 6,848 (43.8%) | 3,024 (38.0%) | 3,824 (49.7%) | 0.90 (0.79-1.01) |
| A level/Degree | 4,401 (28.1%) | 1,966 (24.7%) | 2,435 (31.7%) | 1.30 (1.14-1.48) |
| Missing | 3,167 (20.2%) | 2,439 (30.7%) | 728 (9.5%) |  |
| Financial difficulties in pregnancy | 937 (7.7%) | 439 (8.2%) | 498 (7.3%) | 0.65 (0.56-0.75) |
| Maternal manual occupation | 2,009 (19.9%) | 905 (20.4%) | 1,104 (19.5%) | 0.83 (0.75-0.91) |
| Maternal age at delivery | 28.0 (5.0) | 27.9 (5.1) | 28.1 (4.8) | 1.04 (1.03-1.05) |
| Maternal pre-pregnancy BMI | 22.9 (3.9) | 23.0 (3.9) | 22.9 (3.8) | 0.99 (0.98-1.00) |
|  |  |  |  |  |
| Exposure |  |  |  |  |
| Maternal 25(OH)D (nMol/L) adjusted to 7 weeks ^a^ | 64.1 (32.0) | 63.9 (30.8) | 64.1 (32.0) | 1.04 (1.02-1.06) |
| Maternal 25(OH)D (nMol/L) adjusted to 20 weeks ^a^ | 64.6 (31.6) | 65.2 (29.3) | 64.6 (31.7) | 1.03 (1.02-1.05) |
| Maternal 25(OH)D (nMol/L) adjusted to 34 weeks ^a^ | 69.8 (34.2) | 69.6 (37.2) | 69.8 (34.1) | 1.02 (1.00-1.03) |
| Maternal Standardised Genetic 25(OH)D score | -0.0 (1.0) | 0.0 (1.0) | -0.0 (1.0) | 0.97 (0.93-1.02) |
| Offspring Standardised Genetic 25(OH)D score | 0.0 (1.0) | -0.0 (1.0) | 0.0 (1.0) | 1.03 (0.99-1.08) |
|  |  |  |  |  |
| Outcomes |  |  |  |  |
| Diagnosed Autism | 166 (1.1%) | 73 (1.0%) | 93 (1.2%) | 1.22 (0.90-1.67) |
| Social Communication Trait | 1,385 (17.0%) | 596 (17.1%) | 789 (17.0%) | 0.86 (0.77-0.97) |
| Speech Coherence Trait | 1,284 (15.9%) | 601 (16.3%) | 683 (15.5%) | 0.82 (0.73-0.93) |
| Repetitive Behaviour Trait | 2,177 (27.3%) | 926 (26.8%) | 1,251 (27.6%) | 0.94 (0.85-1.04) |
| Sociability Temperament Trait | 1,148 (11.4%) | 534 (12.3%) | 614 (10.8%) | 0.82 (0.72-0.93) |
| Autism factor mean score | 0.0 (1.0) | 0.0 (1.0) | -0.0 (1.0) | 0.88 (0.85-0.91) |
|  |  |  |  |  |
| Auxiliary variables |  |  |  |  |
| Home ownership status |  |  |  |  |
| Owned/mortgaged | 9,871 (63.1%) | 4,465 (56.1%) | 5,406 (70.3%) | Ref |
| Council rented | 1,939 (12.4%) | 882 (11.1%) | 1,057 (13.7%) | 0.40 (0.36-0.45) |
| Privately rented | 1,204 (7.7%) | 639 (8.0%) | 565 (7.3%) | 0.57 (0.50-0.65) |
| Other | 472 (3.0%) | 224 (2.8%) | 248 (3.2%) | 0.59 (0.48-0.72) |
| Missing | 2,159 (13.8%) | 1,746 (21.9%) | 413 (5.4%) |  |
| Marital status |  |  |  |  |
| Never married | 2,595 (16.6%) | 1,236 (15.5%) | 1,359 (17.7%) | Ref |
| Previously married (currently unmarried) | 816 (5.2%) | 387 (4.9%) | 429 (5.6%) | 1.00 (0.85-1.19) |
| 1st marriage | 9,252 (59.1%) | 4,219 (53.0%) | 5,033 (65.5%) | 1.55 (1.41-1.70) |
| 2nd or 3rd marriage | 881 (5.6%) | 406 (5.1%) | 475 (6.2%) | 1.39 (1.18-1.63) |
| Missing | 2,101 (13.4%) | 1,708 (21.5%) | 393 (5.1%) |  |

^a^ – OR reflects a 10 nmol/L change in Maternal 25(OH)D

## Missing data assessment

Table S3: Missing data descriptives for analyses of the outcomes autism diagnosis, social communication difficulties and speech coherence

|  |  | Diagnosed Autism | | Social communication | | Speech coherence | |
| --- | --- | --- | --- | --- | --- | --- | --- |
| Variable | Total | Complete records | OR (95% CI)^a^ | Complete records | OR (95% CI) ^a^ | Complete records | OR (95% CI) ^a^ |
| Total | N=7,689 | N=5,013 |  | N=3,526 |  | N=3,394 |  |
| Covariates |  |  |  |  |  |  |  |
| Offspring male sex | 4,001 (52.0%) | 2,605 (52.0%) | 0.99 (0.90-1.09) | 1,850 (52.5%) | 1.03 (0.94-1.13) | 1,747 (51.5%) | 0.96 (0.88-1.05) |
| Parity |  |  |  |  |  |  |  |
| 0 | 3,242 (42.2%) | 2,498 (49.8%) | Ref | 1,788 (50.7%) | Ref | 1,717 (50.6%) | Ref |
| 1 | 2,527 (32.9%) | 1,772 (35.3%) | 0.70 (0.62-0.79) | 1,249 (35.4%) | 0.79 (0.72-0.88) | 1,200 (35.4%) | 0.80 (0.72-0.89) |
| 2+ | 1,430 (18.6%) | 743 (14.8%) | 0.32 (0.28-0.37) | 489 (13.9%) | 0.42 (0.37-0.48) | 477 (14.1%) | 0.44 (0.39-0.51) |
| Missing | 490 (6.4%) | 0 (0.0%) |  | 0 (0.0%) |  | 0 (0.0%) |  |
| Any maternal smoking at 18 weeks gestation | 1,935 (26.4%) | 1,079 (21.5%) | 0.46 (0.42-0.52) | 650 (18.4%) | 0.44 (0.40-0.49) | 612 (18.0%) | 0.43 (0.39-0.48) |
| Maternal highest educational qualification |  |  |  |  |  |  |  |
| Vocational | 702 (9.1%) | 486 (9.7%) | Ref | 321 (9.1%) | Ref | 291 (8.6%) | Ref |
| CSE/O level | 3,824 (49.7%) | 2,517 (50.2%) | 0.86 (0.72-1.02) | 1,644 (46.6%) | 0.90 (0.76-1.05) | 1,576 (46.4%) | 0.99 (0.84-1.17) |
| A level/Degree | 2,435 (31.7%) | 2,010 (40.1%) | 2.10 (1.74-2.55) | 1,561 (44.3%) | 2.12 (1.79-2.51) | 1,527 (45.0%) | 2.38 (2.00-2.82) |
| Missing | 728 (9.5%) | 0 (0.0%) |  | 0 (0.0%) |  | 0 (0.0%) |  |
| Financial difficulties in pregnancy | 498 (7.3%) | 299 (6.0%) | 0.51 (0.42-0.62) | 178 (5.0%) | 0.49 (0.41-0.60) | 172 (5.1%) | 0.51 (0.42-0.61) |
| Maternal manual occupation | 1,104 (19.5%) | 928 (18.5%) | 0.62 (0.52-0.75) | 552 (15.7%) | 0.54 (0.47-0.61) | 529 (15.6%) | 0.55 (0.48-0.62) |
| Maternal age at delivery | 28.1 (4.8) | 28.6 (4.6) | 1.07 (1.05-1.08) | 29.1 (4.4) | 1.09 (1.08-1.10) | 29.2 (4.4) | 1.09 (1.08-1.11) |
| Maternal pre-pregnancy BMI | 22.9 (3.8) | 22.8 (3.7) | 0.98 (0.96-0.99) | 22.7 (3.5) | 0.97 (0.96-0.99) | 22.7 (3.5) | 0.98 (0.96-0.99) |
|  |  |  |  |  |  |  |  |
| Exposure |  |  |  |  |  |  |  |
| Maternal 25(OH)D (nMol/L) adjusted to 7 weeks ^b^ | 64.1 (32.0) | 65.5 (32.6) | 1.04 (1.02-1.06) | 65.8 (32.2) | 1.03 (1.02-1.05) | 66.7 (32.6) | 1.05 (1.03-1.06) |
| Maternal 25(OH)D (nMol/L) adjusted to 20 weeks ^b^ | 64.6 (31.7) | 65.8 (32.1) | 1.04 (1.02-1.05) | 66.2 (31.9) | 1.03 (1.02-1.04) | 66.8 (32.2) | 1.04 (1.03-1.05) |
| Maternal 25(OH)D (nMol/L) adjusted to 34 weeks ^b^ | 69.8 (34.1) | 70.5 (34.1) | 1.02 (1.00-1.03) | 71.4 (34.1) | 1.03 (1.01-1.04) | 71.7 (34.0) | 1.03 (1.02-1.04) |
| Maternal Standardised Genetic 25(OH)D score | 0.0 (1.0) | -0.0 (1.0) | 0.97 (0.91-1.03) | -0.0 (1.0) | 0.96 (0.91-1.02) | -0.0 (1.0) | 0.97 (0.91-1.02) |
| Offspring Standardised Genetic 25(OH)D score | 0.0 (1.0) | 0.0 (1.0) | 1.05 (0.98-1.12) | 0.0 (1.0) | 1.03 (0.97-1.09) | 0.0 (1.0) | 1.02 (0.96-1.08) |
|  |  |  |  |  |  |  |  |
| Outcomes |  |  |  |  |  |  |  |
| Diagnosed Autism | 93 (1.2%) | 62 (1.2%) | 1.07 (0.69-1.64) | 55 (1.6%) | 1.72 (1.13-2.60) | 54 (1.6%) | 1.76 (1.16-2.67) |
| Social Communication Trait | 789 (17.0%) | 563 (16.0%) | 0.74 (0.62-0.88) | 563 (16.0%) | 0.74 (0.62-0.88) | 474 (15.6%) | 0.75 (0.64-0.88) |
| Speech Coherence Trait | 683 (15.5%) | 488 (14.4%) | 0.71 (0.59-0.86) | 430 (14.2%) | 0.74 (0.62-0.87) | 488 (14.4%) | 0.71 (0.59-0.86) |
| Repetitive Behaviour Trait | 1,251 (27.6%) | 922 (26.6%) | 0.81 (0.69-0.94) | 784 (26.0%) | 0.79 (0.69-0.91) | 755 (26.1%) | 0.81 (0.71-0.93) |
| Sociability Temperament Trait | 614 (10.8%) | 428 (10.2%) | 0.79 (0.65-0.94) | 348 (10.5%) | 0.92 (0.78-1.09) | 338 (10.5%) | 0.94 (0.79-1.11) |
| Autism factor mean score | -0.0 (1.0) | -0.1 (0.9) | 0.81 (0.77-0.85) | -0.1 (1.0) | 0.85 (0.81-0.90) | -0.1 (1.0) | 0.86 (0.82-0.90) |
|  |  |  |  |  |  |  |  |
| Auxiliary variables |  |  |  |  |  |  |  |
| Home ownership status |  |  |  |  |  |  |  |
| Owned/mortgaged | 5,406 (70.3%) | 4,046 (80.7%) | Ref | 2,987 (84.7%) | Ref | 2,886 (85.0%) | Ref |
| Council rented | 1,057 (13.7%) | 431 (8.6%) | 0.23 (0.20-0.27) | 228 (6.5%) | 0.22 (0.19-0.26) | 212 (6.2%) | 0.22 (0.19-0.26) |
| Privately rented | 565 (7.3%) | 336 (6.7%) | 0.49 (0.41-0.59) | 192 (5.4%) | 0.42 (0.35-0.50) | 188 (5.5%) | 0.44 (0.36-0.52) |
| Other | 248 (3.2%) | 141 (2.8%) | 0.44 (0.34-0.57) | 85 (2.4%) | 0.42 (0.32-0.55) | 77 (2.3%) | 0.39 (0.30-0.52) |
| Missing | 413 (5.4%) | 59 (1.2%) |  | 34 (1.0%) |  | 31 (0.9%) |  |
| Marital status |  |  |  |  |  |  |  |
| Never married | 1,359 (17.7%) | 766 (15.3%) | Ref | 450 (12.8%) | Ref | 424 (12.5%) | Ref |
| Previously married (currently unmarried) | 429 (5.6%) | 240 (4.8%) | 0.98 (0.79-1.22) | 166 (4.7%) | 1.27 (1.02-1.60) | 166 (4.9%) | 1.39 (1.11-1.74) |
| 1st marriage | 5,033 (65.5%) | 3,643 (72.7%) | 2.03 (1.79-2.30) | 2,655 (75.3%) | 2.26 (1.99-2.56) | 2,565 (75.6%) | 2.29 (2.02-2.60) |
| 2nd or 3rd marriage | 475 (6.2%) | 313 (6.2%) | 1.50 (1.20-1.86) | 227 (6.4%) | 1.85 (1.49-2.29) | 214 (6.3%) | 1.81 (1.46-2.24) |
| Missing | 393 (5.1%) | 51 (1.0%) |  | 28 (0.8%) |  | 25 (0.7%) |  |

^a^ – Odds ratio for inclusion in complete record analysis
^b^ – OR reflects a 10 nmol/L change in Maternal 25(OH)D

*Table S4: Missing data descriptives for analyses of the outcomes repetitive behaviour, sociability and autism factor mean score.*

|  |  | Repetitive behaviour | | Sociability temperament | | Autism factor mean score | |
| --- | --- | --- | --- | --- | --- | --- | --- |
| Variable | Total | Complete records | OR (95% CI)^a^ | Complete records | OR (95% CI) ^a^ | Complete records | OR (95% CI) ^a^ |
| Total | N=7,689 | N=3,464 |  | N=4,209 |  | N=4,913 |  |
| Covariates |  |  |  |  |  |  |  |
| Offspring male sex | 4,001 (52.0%) | 1,792 (51.7%) | 0.98 (0.89-1.07) | 2,207 (52.4%) | 1.04 (0.95-1.13) | 2,558 (52.1%) | 1.00 (0.91-1.10) |
| Parity |  |  |  |  |  |  |  |
| 0 | 3,242 (42.2%) | 1,737 (50.1%) | Ref | 2,109 (50.1%) | Ref | 2,441 (49.7%) | Ref |
| 1 | 2,527 (32.9%) | 1,229 (35.5%) | 0.82 (0.74-0.91) | 1,489 (35.4%) | 0.77 (0.69-0.86) | 1,741 (35.4%) | 0.73 (0.65-0.82) |
| 2+ | 1,430 (18.6%) | 498 (14.4%) | 0.46 (0.41-0.53) | 611 (14.5%) | 0.40 (0.35-0.46) | 731 (14.9%) | 0.34 (0.30-0.39) |
| Missing | 490 (6.4%) | 0 (0.0%) |  | 0 (0.0%) |  | 0 (0.0%) |  |
| Any maternal smoking at 18 weeks gestation | 1,935 (26.4%) | 662 (19.1%) | 0.48 (0.43-0.53) | 847 (20.1%) | 0.47 (0.42-0.52) | 1,043 (21.2%) | 0.46 (0.41-0.51) |
| Maternal highest educational qualification |  |  |  |  |  |  |  |
| Vocational | 702 (9.1%) | 298 (8.6%) | Ref | 385 (9.1%) | Ref | 467 (9.5%) | Ref |
| CSE/O level | 3,824 (49.7%) | 1,646 (47.5%) | 1.02 (0.87-1.21) | 2,046 (48.6%) | 0.95 (0.81-1.11) | 2,462 (50.1%) | 0.91 (0.77-1.08) |
| A level/Degree | 2,435 (31.7%) | 1,520 (43.9%) | 2.25 (1.90-2.67) | 1,778 (42.2%) | 2.23 (1.87-2.65) | 1,984 (40.4%) | 2.21 (1.84-2.67) |
| Missing | 728 (9.5%) | 0 (0.0%) |  | 0 (0.0%) |  | 0 (0.0%) |  |
| Financial difficulties in pregnancy | 498 (7.3%) | 183 (5.3%) | 0.54 (0.45-0.65) | 230 (5.5%) | 0.50 (0.42-0.61) | 286 (5.8%) | 0.49 (0.41-0.59) |
| Maternal manual occupation | 1,104 (19.5%) | 561 (16.2%) | 0.59 (0.52-0.68) | 711 (16.9%) | 0.55 (0.48-0.64) | 898 (18.3%) | 0.60 (0.50-0.72) |
| Maternal age at delivery | 28.1 (4.8) | 29.1 (4.4) | 1.09 (1.07-1.10) | 28.9 (4.5) | 1.08 (1.07-1.09) | 28.6 (4.6) | 1.07 (1.06-1.08) |
| Maternal pre-pregnancy BMI | 22.9 (3.8) | 22.8 (3.6) | 0.98 (0.97-1.00) | 22.8 (3.6) | 0.98 (0.97-1.00) | 22.8 (3.7) | 0.98 (0.96-0.99) |
|  |  |  |  |  |  |  |  |
| Exposure |  |  |  |  |  |  |  |
| Maternal 25(OH)D (nMol/L) adjusted to 7 weeks ^b^ | 64.1 (32.0) | 66.1 (32.5) | 1.04 (1.02-1.05) | 66.0 (32.6) | 1.04 (1.03-1.06) | 65.6 (32.6) | 1.04 (1.03-1.06) |
| Maternal 25(OH)D (nMol/L) adjusted to 20 weeks ^b^ | 64.6 (31.7) | 66.2 (31.8) | 1.03 (1.02-1.04) | 66.2 (32.2) | 1.04 (1.02-1.05) | 65.9 (32.1) | 1.04 (1.02-1.05) |
| Maternal 25(OH)D (nMol/L) adjusted to 34 weeks ^b^ | 69.8 (34.1) | 70.9 (34.1) | 1.02 (1.00-1.03) | 70.9 (34.2) | 1.02 (1.01-1.03) | 70.5 (34.1) | 1.02 (1.01-1.03) |
| Maternal Standardised Genetic 25(OH)D score | 0.0 (1.0) | -0.0 (1.0) | 0.99 (0.93-1.05) | -0.0 (1.0) | 0.99 (0.94-1.05) | -0.0 (1.0) | 0.97 (0.91-1.03) |
| Offspring Standardised Genetic 25(OH)D score | 0.0 (1.0) | -0.0 (1.0) | 1.00 (0.94-1.06) | 0.0 (1.0) | 1.01 (0.94-1.07) | 0.0 (1.0) | 1.05 (0.99-1.13) |
|  |  |  |  |  |  |  |  |
| Outcomes |  |  |  |  |  |  |  |
| Diagnosed Autism | 93 (1.2%) | 42 (1.2%) | 1.00 (0.67-1.51) | 57 (1.4%) | 1.31 (0.86-1.99) | 61 (1.2%) | 1.08 (0.70-1.66) |
| Social Communication Trait | 789 (17.0%) | 462 (15.3%) | 0.72 (0.61-0.84) | 532 (16.0%) | 0.78 (0.66-0.92) | 563 (16.0%) | 0.74 (0.62-0.88) |
| Speech Coherence Trait | 683 (15.5%) | 401 (13.9%) | 0.71 (0.60-0.84) | 451 (14.1%) | 0.69 (0.58-0.82) | 488 (14.4%) | 0.71 (0.59-0.86) |
| Repetitive Behaviour Trait | 1,251 (27.6%) | 922 (26.6%) | 0.81 (0.69-0.94) | 873 (26.4%) | 0.79 (0.68-0.91) | 922 (26.6%) | 0.81 (0.69-0.94) |
| Sociability Temperament Trait | 614 (10.8%) | 336 (10.1%) | 0.85 (0.72-1.01) | 428 (10.2%) | 0.79 (0.65-0.94) | 428 (10.2%) | 0.79 (0.65-0.95) |
| Autism Factor Mean Score | -0.0 (1.0) | -0.1 (0.9) | 0.82 (0.78-0.86) | -0.1 (1.0) | 0.82 (0.79-0.87) | -0.1 (0.9) | 0.81 (0.77-0.85) |
|  |  |  |  |  |  |  |  |
| Auxiliary variables |  |  |  |  |  |  |  |
| Home ownership status |  |  |  |  |  |  |  |
| Owned/mortgaged | 5,406 (70.3%) | 2,916 (84.2%) | Ref | 3,497 (83.1%) | Ref | 3,994 (81.3%) | Ref |
| Council rented | 1,057 (13.7%) | 233 (6.7%) | 0.24 (0.21-0.28) | 314 (7.5%) | 0.23 (0.20-0.27) | 406 (8.3%) | 0.22 (0.19-0.25) |
| Privately rented | 565 (7.3%) | 196 (5.7%) | 0.45 (0.38-0.54) | 258 (6.1%) | 0.46 (0.39-0.55) | 319 (6.5%) | 0.46 (0.38-0.55) |
| Other | 248 (3.2%) | 83 (2.4%) | 0.43 (0.33-0.56) | 103 (2.4%) | 0.39 (0.30-0.50) | 137 (2.8%) | 0.44 (0.34-0.56) |
| Missing | 413 (5.4%) | 36 (1.0%) |  | 37 (0.9%) |  | 57 (1.2%) |  |
| Marital status |  |  |  |  |  |  |  |
| Never married | 1,359 (17.7%) | 444 (12.8%) | Ref | 587 (13.9%) | Ref | 737 (15.0%) | Ref |
| Previously married (currently unmarried) | 429 (5.6%) | 168 (4.8%) | 1.33 (1.06-1.66) | 192 (4.6%) | 1.07 (0.86-1.33) | 235 (4.8%) | 1.02 (0.82-1.27) |
| 1st marriage | 5,033 (65.5%) | 2,603 (75.1%) | 2.21 (1.95-2.50) | 3,123 (74.2%) | 2.15 (1.90-2.43) | 3,588 (73.0%) | 2.10 (1.85-2.37) |
| 2nd or 3rd marriage | 475 (6.2%) | 224 (6.5%) | 1.84 (1.49-2.28) | 276 (6.6%) | 1.82 (1.48-2.25) | 307 (6.2%) | 1.54 (1.24-1.91) |
| Missing | 393 (5.1%) | 25 (0.7%) |  | 31 (0.7%) |  | 46 (0.9%) |  |

^a^ – Odds ratio for inclusion in complete record analysis
^b^ – OR reflects a 10 nmol/L change in Maternal 25(OH)D

## Primary analyses repeated with adjustment to 7 weeks and 34 weeks gestation

Table S5: Regression models of each outcome on seasonally and gestational age (7 weeks) adjusted maternal serum 25(OH)D during pregnancy.

|  |  | | Complete record analysis | | Multiple imputation analysis | |
| --- | --- | --- | --- | --- | --- | --- |
| Outcome | Model | Effect estimate | | 95% CI | Effect estimate | 95% CI |
| Autism diagnosis | Unadjusted | | 1.00 | (0.93, 1.08) | 1.00 | (0.94, 1.07) |
|  | Adjusted 1 | | 1.00 | (0.92, 1.08) | 1.01 | (0.94, 1.07) |
|  | Adjusted 2 | | 1.00 | (0.92, 1.08) | 1.00 | (0.94, 1.07) |
|  | Adjusted 3 | | 0.99 | (0.91, 1.07) | 0.99 | (0.93, 1.06) |
|  | Fully adjusted | | 0.99 | (0.91, 1.07) | 0.99 | (0.93, 1.06) |
| Social communication trait | Unadjusted | | 1.01 | (0.98, 1.04) | 1.00 | (0.98, 1.03) |
|  | Adjusted 1 | | 1.00 | (0.98, 1.03) | 1.00 | (0.98, 1.03) |
|  | Adjusted 2 | | 1.01 | (0.98, 1.04) | 1.00 | (0.98, 1.03) |
|  | Adjusted 3 | | 1.01 | (0.99, 1.04) | 1.01 | (0.99, 1.03) |
|  | Fully adjusted | | 1.01 | (0.98, 1.04) | 1.01 | (0.99, 1.04) |
| Speech coherence trait | Unadjusted | | 1.00 | (0.97, 1.03) | 1.00 | (0.98, 1.03) |
|  | Adjusted 1 | | 1.00 | (0.97, 1.03) | 1.01 | (0.98, 1.03) |
|  | Adjusted 2 | | 1.00 | (0.97, 1.03) | 1.01 | (0.98, 1.03) |
|  | Adjusted 3 | | 1.00 | (0.97, 1.03) | 1.01 | (0.98, 1.03) |
|  | Fully adjusted | | 1.00 | (0.97, 1.03) | 1.01 | (0.98, 1.03) |
| Repetitive behaviour trait | Unadjusted | | 0.99 | (0.97, 1.02) | 0.98 | (0.96, 1.00) |
|  | Adjusted 1 | | 0.99 | (0.97, 1.01) | 0.98 | (0.96, 1.00) |
|  | Adjusted 2 | | 0.99 | (0.97, 1.02) | 0.98 | (0.96, 1.00) |
|  | Adjusted 3 | | 1.00 | (0.97, 1.02) | 0.99 | (0.97, 1.01) |
|  | Fully adjusted | | 0.99 | (0.97, 1.02) | 0.99 | (0.97, 1.01) |
| Sociability temperament trait | Unadjusted | | 0.98 | (0.95, 1.01) | 0.98 | (0.96, 1.01) |
|  | Adjusted 1 | | 0.98 | (0.95, 1.01) | 0.98 | (0.96, 1.01) |
|  | Adjusted 2 | | 0.98 | (0.95, 1.01) | 0.98 | (0.96, 1.01) |
|  | Adjusted 3 | | 0.97 | (0.94, 1.01) | 0.98 | (0.95, 1.01) |
|  | Fully adjusted | | 0.97 | (0.94, 1.01) | 0.98 | (0.95, 1.01) |
| Autism Factor Mean Score ^b^ | Unadjusted | | -0.01 | (-0.02, 0.00) | -0.01 | (-0.02, -0.00) |
|  | Adjusted 1 | | -0.01 | (-0.02,-0.00) | -0.01 | (-0.02, -0.00) |
|  | Adjusted 2 | | -0.01 | (-0.01,0.00) | -0.01 | (-0.01, -0.00) |
|  | Adjusted 3 | | -0.01 | (-0.02,0.00) | -0.01 | (-0.02, -0.00) |
|  | Fully adjusted | | -0.01 | (-0.02,0.00) | -0.01 | (-0.02, -0.00) |

Odds ratios reflect a 10 nmol/L change in adjusted vitamin D
Adjusted 1 = adjusted for offspring sex;
Adjusted 2 = adjusted for financial difficulties, maternal education, and maternal occupational class;
Adjusted 3 = adjusted for parity, maternal age at birth, pre-pregnancy BMI and smoking status during pregnancy;
Fully adjusted = adjusted for all variables in adjusted 1-3.
Complete record analysis N = 5013 for autism diagnosis; 3526 for social communication trait; 3394 for speech coherence trait; 3464 for repetitive behaviour trait; 4209 for sociability temperament trait; 4913 for autism factor mean score.
Multiple imputation analysis N = 7686 for all outcomes.

Table S6: Regression models of each outcome on seasonally and gestational age (34 weeks) adjusted maternal serum 25(OH)D during pregnancy.

|  |  | Complete record analysis | | Multiple imputation analysis | |
| --- | --- | --- | --- | --- | --- |
| Outcome | Model | Effect estimate | 95% CI | Effect estimate | 95% CI |
| Autism diagnosis ^a^ | Unadjusted | 1.00 | (0.93, 1.08) | 1.01 | (0.95, 1.07) |
|  | Adjusted 1 | 1.00 | (0.93, 1.08) | 1.01 | (0.95, 1.07) |
|  | Adjusted 2 | 1.00 | (0.93, 1.08) | 1.00 | (0.95, 1.06) |
|  | Adjusted 3 | 0.99 | (0.92, 1.07) | 1.00 | (0.94, 1.06) |
|  | Fully adjusted | 0.99 | (0.92, 1.07) | 1.00 | (0.94, 1.06) |
| Social communication trait ^a^ | Unadjusted | 0.99 | (0.96, 1.02) | 0.99 | (0.97, 1.01) |
|  | Adjusted 1 | 0.99 | (0.96, 1.01) | 0.99 | (0.97, 1.01) |
|  | Adjusted 2 | 0.99 | (0.96, 1.02) | 0.99 | (0.97, 1.02) |
|  | Adjusted 3 | 1.00 | (0.97, 1.02) | 1.00 | (0.98, 1.02) |
|  | Fully adjusted | 0.99 | (0.97, 1.02) | 1.00 | (0.98, 1.02) |
| Speech coherence trait ^a^ | Unadjusted | 0.98 | (0.95, 1.01) | 0.99 | (0.96, 1.01) |
|  | Adjusted 1 | 0.98 | (0.95, 1.01) | 0.99 | (0.96, 1.01) |
|  | Adjusted 2 | 0.98 | (0.96, 1.01) | 0.99 | (0.97, 1.01) |
|  | Adjusted 3 | 0.98 | (0.95, 1.01) | 0.99 | (0.97, 1.01) |
|  | Fully adjusted | 0.98 | (0.95, 1.01) | 0.99 | (0.97, 1.01) |
| Repetitive behaviour trait ^a^ | Unadjusted | 0.98 | (0.96, 1.00) | 0.97 | (0.96, 0.99) |
|  | Adjusted 1 | 0.98 | (0.96, 1.00) | 0.97 | (0.96, 0.99) |
|  | Adjusted 2 | 0.98 | (0.96, 1.00) | 0.98 | (0.96, 0.99) |
|  | Adjusted 3 | 0.98 | (0.96, 1.01) | 0.98 | (0.96, 1.00) |
|  | Fully adjusted | 0.98 | (0.96, 1.01) | 0.98 | (0.96, 1.00) |
| Sociability temperament trait ^a^ | Unadjusted | 1.00 | (0.97, 1.03) | 1.00 | (0.98, 1.02) |
|  | Adjusted 1 | 1.00 | (0.97, 1.03) | 1.00 | (0.98, 1.02) |
|  | Adjusted 2 | 1.00 | (0.98, 1.03) | 1.00 | (0.98, 1.03) |
|  | Adjusted 3 | 1.00 | (0.97, 1.03) | 1.00 | (0.98, 1.02) |
|  | Fully adjusted | 1.00 | (0.97, 1.03) | 1.00 | (0.98, 1.03) |
| Autism Factor Mean Score ^b^ | Unadjusted | -0.01 | (-0.01, 0.00) | -0.01 | (-0.02, -0.00) |
|  | Adjusted 1 | -0.01 | (-0.01, 0.00) | -0.01 | (-0.01, -0.00) |
|  | Adjusted 2 | 0.00 | (-0.01, 0.00) | -0.01 | (-0.01, -0.00) |
|  | Adjusted 3 | -0.01 | (-0.01, 0.00) | -0.01 | (-0.01, -0.00) |
|  | Fully adjusted | -0.01 | (-0.01, 0.00) | -0.01 | (-0.01, -0.00) |

^a^ – effect estimate = odds ratio
^b^ – effect estimate = mean difference
Effect estimates reflect a 10 nmol/L change in adjusted vitamin D
Adjusted 1 = adjusted for offspring sex;
Adjusted 2 = adjusted for financial difficulties, maternal education, and maternal occupational class;
Adjusted 3 = adjusted for parity, maternal age at birth, pre-pregnancy BMI and smoking status during pregnancy;
Fully adjusted = adjusted for all variables in adjusted 1-3.
Complete record analysis N = 5013 for autism diagnosis; 3526 for social communication trait; 3394 for speech coherence trait; 3464 for repetitive behaviour trait; 4209 for sociability temperament trait; 4913 for autism factor mean score.
Multiple imputation analysis N = 7686 for all outcomes.

## Primary analyses restricted to individuals of European ancestry

Table S7: Sensitivity analysis regression models of each outcome on maternal 25(OH)D adjusted for season and to 20 weeks gestation among the sample with European ancestry as determined by genetic information.

|  |  | Complete record analysis | | Multiple imputation analysis | |
| --- | --- | --- | --- | --- | --- |
| Outcome | Model | Effect estimate | 95% CI | Effect estimate | 95% CI |
| Autism diagnosis ^a^ | Unadjusted | 0.93 | (0.83, 1.04) | 0.97 | (0.89, 1.06) |
|  | Adjusted 1 | 0.93 | (0.83, 1.03) | 0.97 | (0.89, 1.06) |
|  | Adjusted 2 | 0.93 | (0.83, 1.03) | 0.97 | (0.89, 1.05) |
|  | Adjusted 3 | 0.92 | (0.82, 1.03) | 0.96 | (0.87, 1.04) |
|  | Fully adjusted | 0.92 | (0.82, 1.03) | 0.96 | (0.87, 1.04) |
| Social communication trait ^a^ | Unadjusted | 0.98 | (0.94, 1.01) | 0.98 | (0.95, 1.01) |
|  | Adjusted 1 | 0.98 | (0.94, 1.01) | 0.98 | (0.95, 1.01) |
|  | Adjusted 2 | 0.98 | (0.95, 1.01) | 0.98 | (0.95, 1.01) |
|  | Adjusted 3 | 0.98 | (0.95, 1.02) | 0.98 | (0.95, 1.01) |
|  | Fully adjusted | 0.98 | (0.95, 1.02) | 0.98 | (0.95, 1.01) |
| Speech coherence trait ^a^ | Unadjusted | 0.97 | (0.94, 1.01) | 1.00 | (0.97, 1.03) |
|  | Adjusted 1 | 0.97 | (0.94, 1.01) | 1.00 | (0.97, 1.03) |
|  | Adjusted 2 | 0.98 | (0.94, 1.01) | 1.00 | (0.97, 1.03) |
|  | Adjusted 3 | 0.97 | (0.94, 1.01) | 1.00 | (0.97, 1.03) |
|  | Fully adjusted | 0.98 | (0.94, 1.01) | 1.00 | (0.97, 1.03) |
| Repetitive behaviour trait ^a^ | Unadjusted | 0.98 | (0.95, 1.01) | 0.97 | (0.94, 0.99) |
|  | Adjusted 1 | 0.98 | (0.95, 1.00) | 0.97 | (0.94, 0.99) |
|  | Adjusted 2 | 0.98 | (0.95, 1.01) | 0.97 | (0.94, 0.99) |
|  | Adjusted 3 | 0.98 | (0.95, 1.01) | 0.97 | (0.95, 1.00) |
|  | Fully adjusted | 0.98 | (0.95, 1.01) | 0.97 | (0.95, 1.00) |
| Sociability temperament trait ^a^ | Unadjusted | 1.00 | (0.96, 1.03) | 0.99 | (0.96, 1.03) |
|  | Adjusted 1 | 0.99 | (0.96, 1.03) | 0.99 | (0.96, 1.03) |
|  | Adjusted 2 | 1.00 | (0.96, 1.03) | 0.99 | (0.96, 1.03) |
|  | Adjusted 3 | 0.99 | (0.95, 1.03) | 0.99 | (0.96, 1.02) |
|  | Fully adjusted | 0.99 | (0.95, 1.03) | 0.99 | (0.96, 1.02) |
|  |  |  |  |  |  |
| Autism Factor Mean Score ^b^ | Unadjusted | -0.01 | (-0.02, -0.00) | -0.01 | (-0.02, -0.00) |
|  | Adjusted 1 | -0.01 | (-0.02, -0.00) | -0.01 | (-0.02, -0.00) |
|  | Adjusted 2 | -0.01 | (-0.02, -0.00) | -0.01 | (-0.02, -0.00) |
|  | Adjusted 3 | -0.01 | (-0.02, -0.00) | -0.01 | (-0.02, -0.00) |
|  | Fully adjusted | -0.01 | (-0.02, -0.00) | -0.01 | (-0.02, -0.00) |

^a^ – effect estimate = odds ratio
^b^ – effect estimate = mean difference
Effect estimates reflect a 10 nmol/L change in adjusted vitamin D
Adjusted 1 = adjusted for offspring sex;
Adjusted 2 = adjusted for financial difficulties, maternal education, and maternal occupational class;
Adjusted 3 = adjusted for parity, maternal age at birth, pre-pregnancy BMI and smoking status during pregnancy;
Fully adjusted = adjusted for all variables in adjusted 1-3.
Note that the auxiliary variables marital status and home ownership were removed from the imputation model due to issues of perfect prediction in the European ancestry sample.
Complete record analysis N = 3088 for autism diagnosis; 2455 for social communication trait; 2361 for speech coherence trait; 2394 for repetitive behaviour trait; 2848 for sociability temperament trait; 3235 for autism factor mean score.
Multiple imputation analysis N = 4718 for all outcomes.

## Sensitivity of spline analyses to the number of knots used


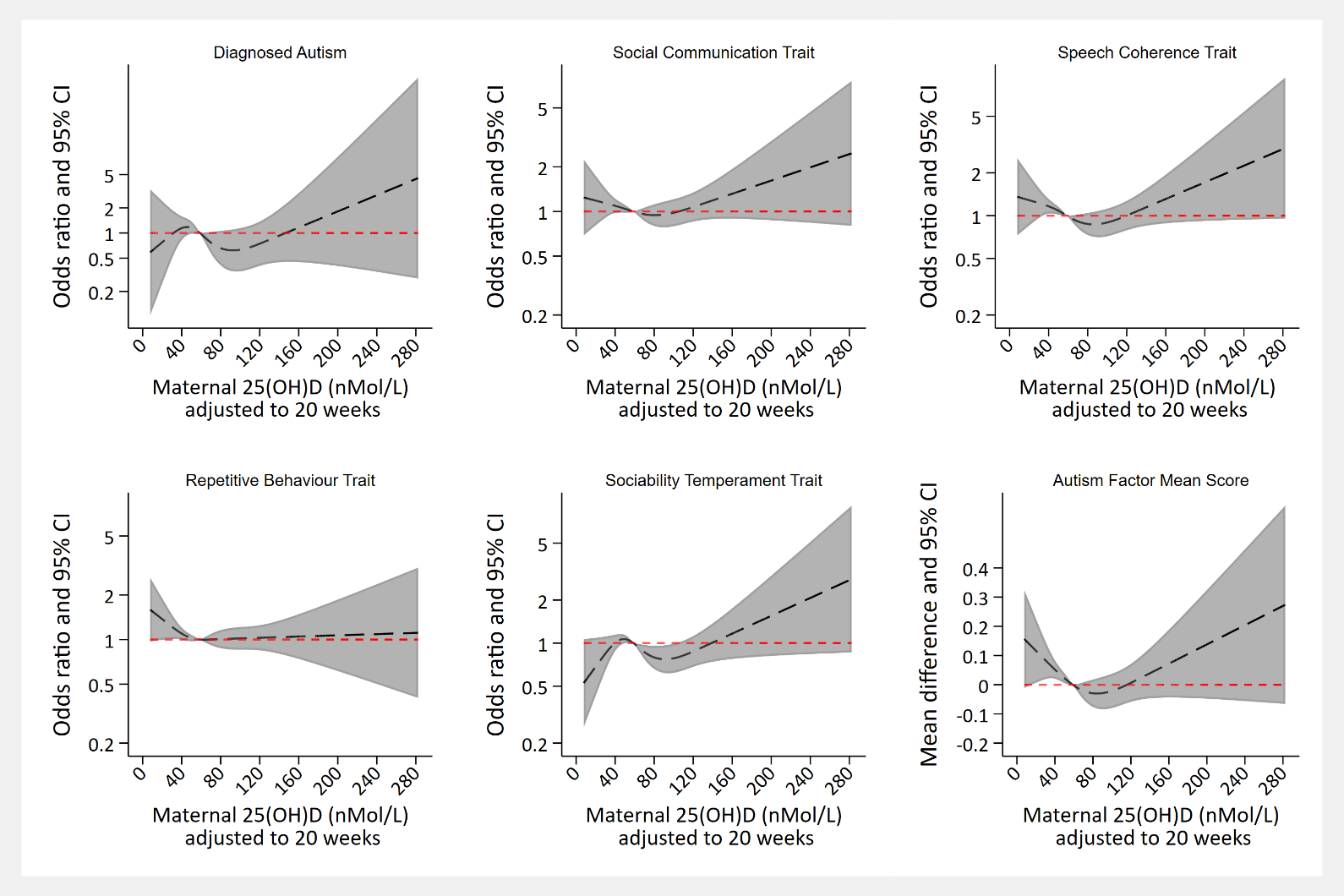


Figure S3: Sensitivity analyses of spline models using 4 knots. Regression of each outcome on maternal 25(OH)D adjusted to 20 weeks using restricted cubic regression splines with five knots in order to explore non-linear associations between exposure and outcome. Model adjusted for parity, offspring sex, maternal age at birth, pre-pregnancy BMI, smoking status during pregnancy, financial difficulties during pregnancy, maternal education and maternal occupational class


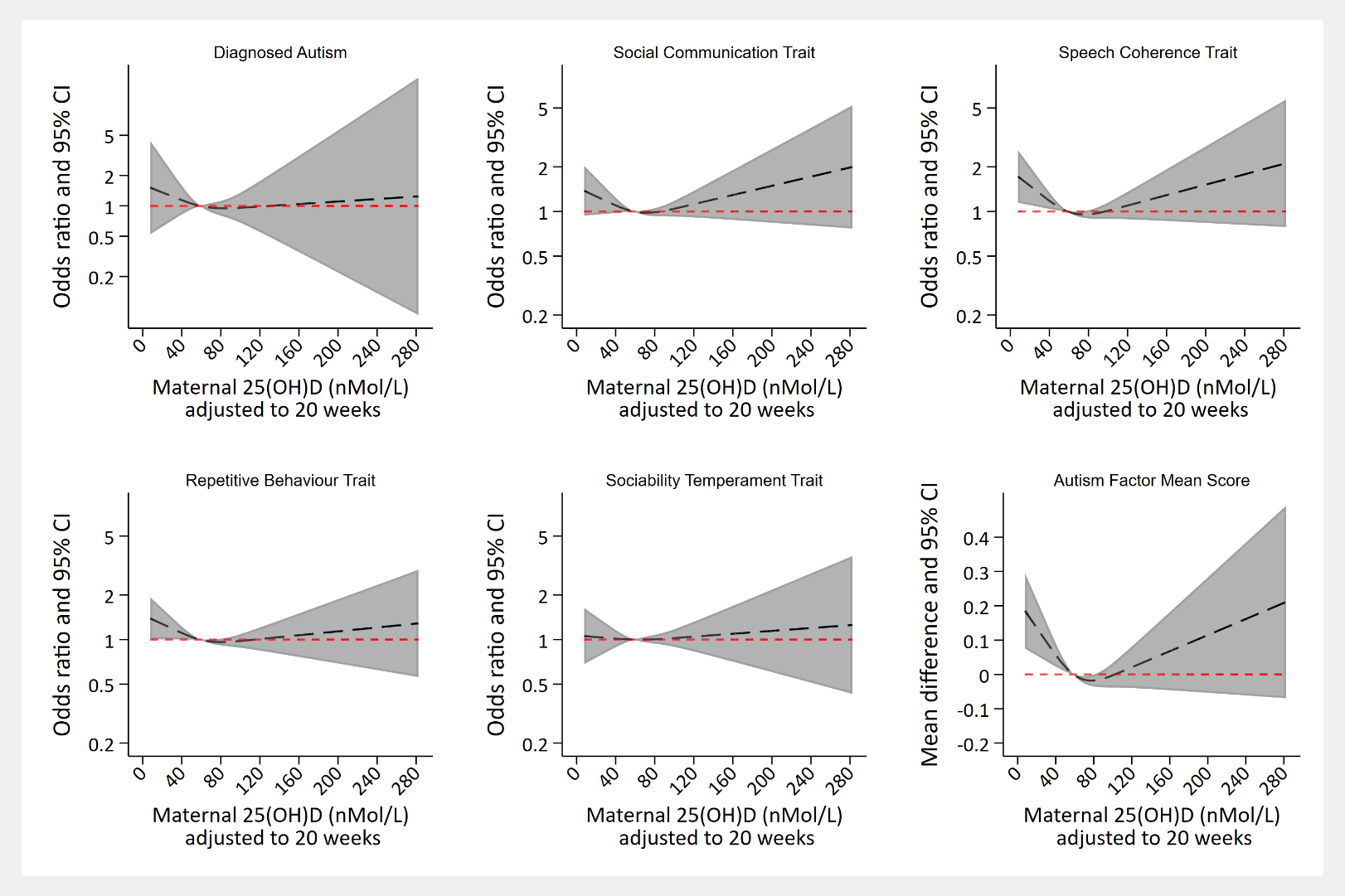


Figure S4: Sensitivity analyses of spline models using 3 knots. Regression of each outcome on maternal 25(OH)D adjusted to 20 weeks using restricted cubic regression splines with five knots in order to explore non-linear associations between exposure and outcome. Model adjusted for parity, offspring sex, maternal age at birth, pre-pregnancy BMI, smoking status during pregnancy, financial difficulties during pregnancy, maternal education and maternal occupational class.

## MR Sensitivity analysis

Table S8: Association between maternal genetic risk score for vitamin D and each confounder variable using separate linear regression models with no adjustment to explore the presence of horizontal pleiotropy

| Variable | Level | Mean difference in maternal genetic risk score for vitamin D | R^2^ |
| --- | --- | --- | --- |
| Sex | Female | Ref | <0.001 |
|  | Male | -0.00 (-0.06, 0.05) |  |
| Parity | 0 | Ref | <0.001 |
|  | 1 | 0.01 (-0.06, 0.07) |  |
|  | 2+ | -0.02 (-0.10, 0.06) |  |
| Maternal smoking in pregnancy | No | Ref | <0.001 |
|  | Yes | 0.03 (-0.04, 0.10) |  |
| Maternal education | Vocational | Ref | <0.001 |
|  | CSE/O level | -0.03 (-0.14, 0.08) |  |
|  | A level/Degree | -0.01 (-0.12, 0.09) |  |
| Financial difficulties | No | Ref | <0.001 |
|  | Yes | -0.07 (-0.19, 0.05) |  |
| Maternal occupation | Non-manual | Ref | 0.001 |
|  | Manual | -0.07 (-0.15, 0.02) |  |
| Maternal age |  | -0.00 (-0.01, 0.00) | <0.001 |
| Pre-pregnancy BMI |  | 0.00 (-0.00, 0.01) | <0.001 |

# References

1. McAree T, Jacobs B, Manickavasagar T, Sivalokanathan S, Brennan L, Bassett P, et al. Vitamin D deficiency in pregnancy - still a public health issue. Matern Child Nutr. 2013;9(1):23-30.

2. Gardener H, Spiegelman D, Buka SL. Prenatal risk factors for autism: comprehensive meta-analysis. Br J Psychiatry. 2009;195(1):7-14.

3. Sutherland JP, Zhou A, Leach MJ, Hypponen E. Differences and determinants of vitamin D deficiency among UK biobank participants: A cross-ethnic and socioeconomic study. Clin Nutr. 2021;40(5):3436-47.

4. Williams E, Thomas K, Sidebotham H, Emond A. Prevalence and characteristics of autistic spectrum disorders in the ALSPAC cohort. Dev Med Child Neurol. 2008;50(9):672-7.

5. Karlsson T, Andersson L, Hussain A, Bosaeus M, Jansson N, Osmancevic A, et al. Lower vitamin D status in obese compared with normal-weight women despite higher vitamin D intake in early pregnancy. Clin Nutr. 2015;34(5):892-8.

6. Andersen CH, Thomsen PH, Nohr EA, Lemcke S. Maternal body mass index before pregnancy as a risk factor for ADHD and autism in children. Eur Child Adolesc Psychiatry. 2018;27(2):139-48.

7. Brot C, Jorgensen NR, Sorensen OH. The influence of smoking on vitamin D status and calcium metabolism. Eur J Clin Nutr. 1999;53(12):920-6.

8. Kalkbrenner AE, Meier SM, Madley-Dowd P, Ladd-Acosta C, Fallin MD, Parner E, et al. Familial confounding of the association between maternal smoking in pregnancy and autism spectrum disorder in offspring. Autism Res. 2020;13(1):134-44.

9. Lehti V, Hinkka-Yli-Salomaki S, Cheslack-Postava K, Gissler M, Brown AS, Sourander A. Maternal socio-economic status based on occupation and autism spectrum disorders: a national case-control study. Nord J Psychiatry. 2015;69(7):523-30.

10. Office of population censuses and surveys, General register office for Scotland. 1991 Census: Definitions Great Britain. London: HMSO; 1992.

11. Fraser A, Macdonald-Wallis C, Tilling K, Boyd A, Golding J, Davey Smith G, et al. Cohort Profile: the Avon Longitudinal Study of Parents and Children: ALSPAC mothers cohort. Int J Epidemiol. 2013;42(1):97-110.

12. Paternoster L, Evans DM, Nohr EA, Holst C, Gaborieau V, Brennan P, et al. Genome-wide population-based association study of extremely overweight young adults--the GOYA study. PLoS One. 2011;6(9):e24303.

13. The 1000 Genomes Project Consortium, Abecasis GR, Auton A, Brooks LD, DePristo MA, Durbin RM, et al. An integrated map of genetic variation from 1,092 human genomes. Nature. 2012;491(7422):56-65.

14. Manousaki D, Mitchell R, Dudding T, Haworth S, Harroud A, Forgetta V, et al. Genome-wide Association Study for Vitamin D Levels Reveals 69 Independent Loci. Am J Hum Genet. 2020;106(3):327-37.

15. Bycroft C, Freeman C, Petkova D, Band G, Elliott LT, Sharp K, et al. The UK Biobank resource with deep phenotyping and genomic data. Nature. 2018;562(7726):203-9.

16. Purcell S. PLINK (v1.90) [Available from: <http://pngu.mgh.harvard.edu/purcell/plink/>.

17. Purcell S, Neale B, Todd-Brown K, Thomas L, Ferreira MAR, Bender D, et al. PLINK: A tool set for whole-genome association and population-based linkage analyses. Am J Hum Genet. 2007;81(3):559-75.
